# Supplementary material for: A blueprint for a synthetic genetic feedback optimizer
Source: Nat Commun. 2023 May 3;14:2554. doi: 10.1038/s41467-023-37903-0 (PMC10156725; doi:10.1038/s41467-023-37903-0)
Supplement: Supplementary file 1 — Supplementary Information [file 41467_2023_37903_MOESM1_ESM.pdf]

A blueprint for a synthetic genetic feedback optimizer

Supplementary Information

Andras Gyorgy,[1*] Amor Menezes,[2] Murat Arcak[3]

[1]Division of Engineering, New York University Abu Dhabi, Abu Dhabi, UAE
[2]Department of Mechanical and Aerospace Engineering,
University of Florida, Gainesville, FL USA
[3]Department of Electrical Engineering and Computer Sciences,
University of California, Berkeley, CA USA

[*]To whom correspondence should be addressed; E-mail: andras.gyorgy@nyu.edu.

This Supplementary Information includes details on the (i) dynamics and behavior of the simplified optimizer with the three main modules comprising it; (ii) concrete molecular implementation of the whole integrated system together with the corresponding mass action kinetics-based dynamics and parameter values typical in *E. coli*; (iii) mathematical models of the application examples; (iv) estimated bioenergetic cost of the optimizer; and (v) simulation data presented in the article.

Contents

1 Dynamics and behavior of the simplified optimizer . . . 2

2 Implementation based on existing synthetic biology parts and components . . . 13

3 Application examples . . . 26

4 Metabolic burden . . . 36

5 Simulation details . . . 40

1 Dynamics and behavior of the simplified optimizer

1.1 Regulator dynamics

Consider the scalar dynamics $\dot{X} = \alpha - \gamma X$ such that α and γ are the production and decay rate constants of species X. By changing these two parameters, various effects of context-dependence can be modeled, for instance, competition for shared transcriptional/translational (*1–3*) and degradation (*4*) resources decrease α and γ, respectively. Next, assume that we can regulate the effective production and decay rates of X via the control signals $0 \leq u_1, u_2 \leq 1$ according to $\dot{X} = \alpha\lambda_\alpha u_1 - \gamma\lambda u_2 X$, where $\lambda_\alpha, \lambda \geq 0$, which can be rewritten with

$$\epsilon_x = \frac{1}{\gamma\lambda}, \qquad x = \frac{X}{\epsilon_x \alpha \lambda_\alpha} \tag{1}$$

as $\epsilon_x \dot{x} = u_1 - u_2 x$, the dynamics that we consider in the article. Note that ϵ_x can be further eliminated via rescaling time as $t \leftarrow t/\epsilon_x$.

Here, we consider the optimizer structure from Fig. 1c where the delay, comparator, and logic modules are implemented as follows. Regarding the delay module, let $x_d(t) = x(t - t_d)$ and $y_d(t) = y(t - t_d)$ for $t_d > 0$. Within the comparator module, we have $x_+ = h(x - x_d)$ and $x_- = h(x_d - x)$ such that $h(w) = 1$ for $w > 0$ and $h(w) = 0$ otherwise. The indicator signals $y_+, y_- \geq 0$ are defined similarly. Finally, the control law is implemented by the logic module as $u_1 = x_+ y_+ + x_- y_-$ and $u_2 = x_+ y_- + x_- y_+$.

Supplementary Fig. 1 presents the closed loop performance with the objective function

$$F(x, \theta_y) = F_0 e^{-\frac{(x - x^*)^2}{2\sigma^2}}, \tag{2}$$

where x^* is the (time-varying) optimum and σ characterizes how rapidly performance drops as we depart from the optimum location. Unless otherwise stated, we consider $F_0 = 1$ and $\epsilon_x = 100$, but the results can be easily generalized for different values of ϵ_x by rescaling time t as $t \leftarrow t/\epsilon_x$ and $\epsilon_y \leftarrow \epsilon_y/\epsilon_x$, and similarly for different values of F_0 by rescaling y as $y \rightarrow F_0 y$.

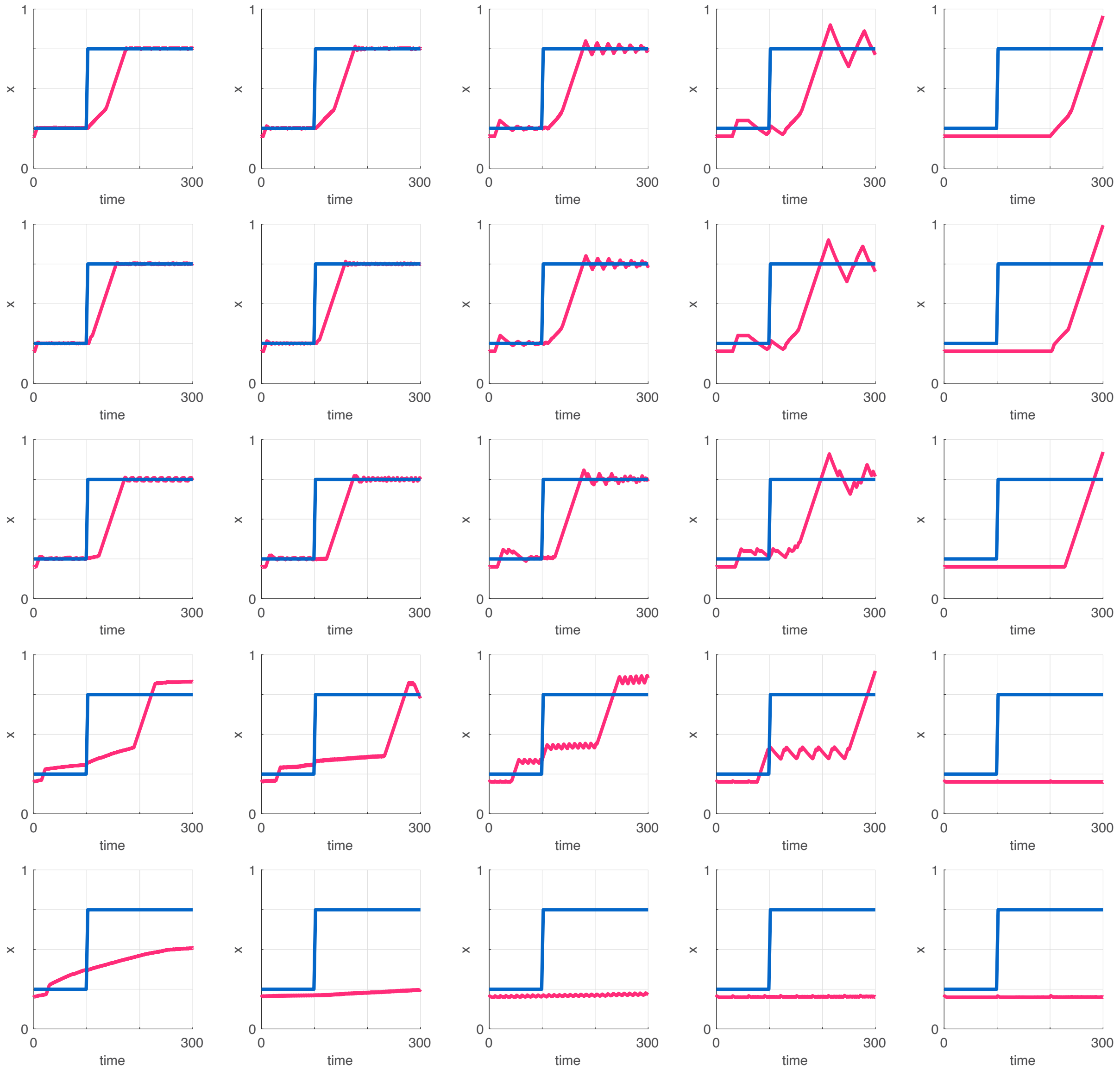

Supplementary Fig. 1. **Effects of ϵ_y and t_d on the closed loop performance.** From top to bottom ϵ_y changes as 0.01, 0.1, 1, 10, 100, and from left to right t_d changes as 1, 3, 10, 30, 100. In all plots $\sigma = x^*/10$. Blue curve denotes the time-varying location of the optimum.

1.2 Delay module

Consider species X driving the expression of X_d, given by the dynamics

$$\frac{\mathrm{d}X_d}{\mathrm{d}t} = \frac{\alpha_{X_d} X}{K_{X_d} + X} - \gamma_d X_d,$$

where α_{X_d}, γ_d, and K_{X_d} are the corresponding production, decay, and dissociation rate constants, respectively. From (1) it follows that

$$\epsilon_d \dot{x}_d = \frac{\alpha_{x_d}}{1 + x\frac{\epsilon_x \alpha \lambda_\alpha}{K_{X_d}}} x - x_d$$

with

$$x_d = \frac{X_d}{K_{X_d}}, \qquad \epsilon_d = \frac{1}{\gamma_d}, \qquad \alpha_{x_d} = \frac{\alpha_{X_d} \epsilon_x \alpha \lambda_\alpha}{\gamma_d K_{X_d}^2}. \tag{3}$$

Therefore, the dynamics of x_d simplifies to $\epsilon_d \dot{x}_d = \alpha_{x_d} x - x_d$ if $K_{X_d} \gg x \epsilon_x \alpha \lambda_\alpha$. A similar rescaling can be performed for the dynamics of y_d, yielding $\epsilon_d \dot{y}_d = \alpha_{y_d} y - y_d$.

While we ideally seek $\alpha_{x_d} = \alpha_{y_d} = 1$, such precision cannot be guaranteed. To account for this imprecision and for the effects of the denominator in (3), we assume that $\alpha_{x_d}, \alpha_{y_d} \approx 1$. We show in Fig. 5b that the optimizer works even when considering a non-ideal implementation. As expected, closed loop performance quickly degrades as the timescale of either the sensor or the delay module approaches that of the regulator (Supplementary Fig. 2).

1.3 Comparator module

Here, we focus on the comparator with species X_+ and X_- and the periodic signal c (Fig. 3a). After presenting the underlying dynamics during both phases (corresponding to $c = 0$ and $c = 1$), we focus on the response time and dynamic range of the module, together with how unbalancedness affects performance and how its adverse affects can be mitigated.

Dynamics

During phase 1 ($c = 0$), the dynamics are given by

$$\dot{X}_+ = \frac{\bar{\alpha}_{X_+} X}{K_X + X} - \gamma_c X_+, \qquad \dot{X}_- = \frac{\bar{\alpha}_{X_-} X_d}{K_{X_d} + X_d} - \gamma_c X_-, \tag{4}$$

where $\bar{\alpha}_{X_+}$ and $\bar{\alpha}_{X_-}$ are production rate constants; K_X and K_{X_d} are dissociation rate constants; and γ_c denotes the decay rate. During phase 2 ($c = 1$), the system behaves as a toggle switch (*5*)

with dynamics

$$\dot{X}_+ = \frac{\alpha_{X_+}}{K_{X_-}^n + X_-^n} - \gamma_c X_+, \qquad \dot{X}_- = \frac{\alpha_{X_-}}{K_{X_+}^n + X_+^n} - \gamma_c X_-, \tag{5}$$

where α_{X_+} and α_{X_-} are production rate constants; K_{X_+} and K_{X_-} are dissociation rate constants; n denotes the Hill coefficient of the repressors; and γ_c is the decay rate constant.

Introduce

$$\epsilon_c = \frac{1}{\gamma_c}, \quad x_+ = \frac{X_+}{K_{X_+}}, \quad x_- = \frac{X_-}{K_{X_-}}, \quad \alpha_{x_+} = \frac{\alpha_{X_+}}{\gamma_c K_{X_+} K_{X_-}^n}, \quad \alpha_{x_-} = \frac{\alpha_{X_-}}{\gamma_c K_{X_+}^n K_{X_-}},$$

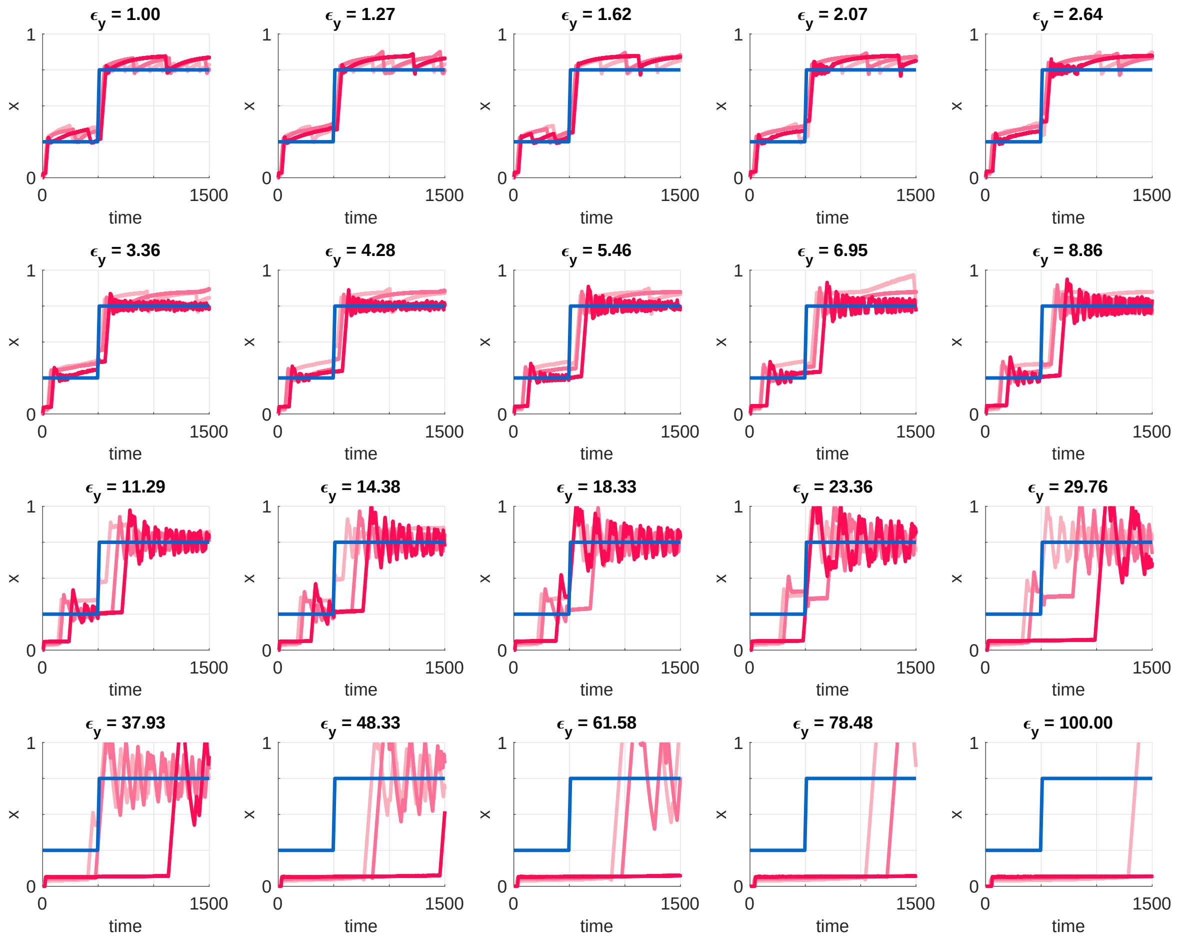

Supplementary Fig. 2. **Effects of ϵ_y and ϵ_d approaching ϵ_x.** In all plots $\epsilon_x = 100$. Light, medium, and dark red correspond to $\epsilon_d = \epsilon_y/2$, $\epsilon_d = \epsilon_y$, and $\epsilon_d = 2\epsilon_y$, respectively. Blue curve denotes the time-varying location of the optimum.

so that the phase 1 and phase 2 dynamics in (4)–(5) can be written as

$$\begin{aligned}
\text{phase 1:}\quad & \epsilon_c \dot{x}_+ = \frac{\bar{\alpha}_{x_+}}{1 + x\frac{\epsilon_x \alpha \lambda_\alpha}{K_X}} x - x_+, && \epsilon_c \dot{x}_- = \frac{\bar{\alpha}_{x_-}}{1 + x_d\frac{\epsilon_x \alpha \lambda_\alpha}{K_{X_d}}} x_d - x_-, \\
\text{phase 2:}\quad & \epsilon_c \dot{x}_+ = \frac{\alpha_{x_+}}{1 + x_-^n} - x_+, && \epsilon_c \dot{x}_- = \frac{\alpha_{x_-}}{1 + x_+^n} - x_-,
\end{aligned} \tag{6}$$

with

$$\bar{\alpha}_{x_+} = \frac{\bar{\alpha}_{X_+}\epsilon_x \alpha \lambda_\alpha}{\gamma_c K_X K_{X_+}}, \qquad \bar{\alpha}_{x_-} = \frac{\bar{\alpha}_{X_-}\epsilon_x \alpha \lambda_\alpha}{\gamma_c K_{X_d} K_{X_-}}.$$

Summarizing the dynamics during phase 1 and phase 2 with the periodic signal c such that the former is activated when $c = 0$ and the latter when $c = 1$, we can thus write (6) as

$$\begin{aligned}
\epsilon_c \dot{x}_+ =& (1-c)\left(\bar{\alpha}_{x_+} x - x_+\right) + c\left(\frac{\alpha_{x_+}}{1 + x_-^n} - x_+\right), \\
\epsilon_c \dot{x}_- =& (1-c)\left(\bar{\alpha}_{x_-} x - x_-\right) + c\left(\frac{\alpha_{x_-}}{1 + x_+^n} - x_-\right).
\end{aligned}$$

provided that $x \ll K_X/(\epsilon_x \alpha \lambda_\alpha)$ and $x_d \ll K_{X_d}/(\epsilon_x \alpha \lambda_\alpha)$.

In what follows, we assume that $n = 2$, i.e., repressors act as dimers (*6–12*). However, this restriction can be easily removed to extend the results to more general cases (*13–18*). Furthermore, we assume that $\bar{\alpha}_{x_-} = \bar{\alpha}_{x_+} = \alpha_{c,1} = 1$, yielding perfect tracking with no steady state error in phase 1; the effects of imperfect tracking are illustrated in Fig. 5b. Finally, we first focus on a balanced realization with $\alpha_{c,2} = \alpha_{x_-} = \alpha_{x_+}$, but then relax this assumption (as in Fig. 5c) to reveal the adverse effects of imbalance and how to mitigate them.

Response time and dynamic range

In phase 1, both x_+ and x_- evolve according to independent scalar linear ordinary differential equations with inputs x and x_d. Furthermore, when $\epsilon_c \ll \epsilon_x, \epsilon_d$, the dynamics of x_+ and x_- are much faster than that of x and x_d. Hence, on this fast timescale t/ϵ_c, the latter are effectively "frozen" at their values in the beginning of phase 1. Consequently, x_+ and x_- converge to these initial values with a time constant $1/\epsilon_c$, so that over a $5/\epsilon_c$ period they effectively converge to the "frozen" values of x and x_d, respectively.

In phase 2, these "frozen" values then serve as initial conditions for the dynamics in (6). Introduce $z = (x_+, x_-)$. The system is bistable if $\alpha_{c,2} > 2$ with the stable fixed points located at $z_+ = (\bar{s}, \underline{s})$ and $z_- = (\underline{s}, \bar{s})$ where $\underline{s} = (\alpha_{c,2} - \sqrt{\alpha_{c,2}^2 - 4})/2$ and $\bar{s} = (\alpha_{c,2} + \sqrt{\alpha_{c,2}^2 - 4})/2$. If the "frozen" value of x exceeds that of x_d at the start of phase 1, then trajectories converge to the x_+-dominated stable fixed point z_+, otherwise they converge to the x_--dominated stable equilibrium z_-. Hence, the dynamic range of the comparator is given by $\bar{s}/\underline{s}$.

To characterize the time that it takes to reach one of the stable equilibria in phase 2, define

$$T_{SET}(\delta) = \max_{0 \leq x_+(0), x_-(0) \leq \alpha_{c,2}} \inf \left\{ t \mid \min\left(d_+(t), d_-(t)\right) < \delta \right\},$$

where $d_+(t) = \|z(t) - z_+\|_2$ and $d_-(t) = \|z(t) - z_-\|_2$ denote the distance from the x_+-dominated and x_--dominated stable equilibria, respectively. Therefore, $T_{\text{SET}}(\delta)$ measures the time it takes to set the toggle switch with a given threshold. For instance, to get within 5% of one the stable fixed points, it takes approximately $t = 20\epsilon_c$ if $\alpha_{c,2} > 10$, independent of the value of $\alpha_{c,2}$ (Supplementary Fig. 3a).

Parameter asymmetry

Consider the general case of an unbalanced toggle switch (i.e., $\alpha_{x_+} \neq \alpha_{x_-}$), and without loss of generality, assume that $\alpha_{x_+} > \alpha_{x_-}$. In this case, it is not necessarily true that if the "frozen" value of x_d exceeds that of x then trajectories in phase 2 converge to the x_+-dominated stable fixed point of (5), as illustrated in Supplementary Fig. 3b.

To characterize this adverse effect, let (x_+^∞, x_-^∞) denote the stable fixed point toward which a trajectory of (5) converges from a given initial condition (x_+^0, x_-^0) at $t = 0$, and define $\Psi = \left\{(x_+^0, x_-^0) \mid (x_+^0 - x_-^0)(x_+^\infty - x_-^\infty) < 0\right\}$, that is, the set of initial conditions that yield convergence to the incorrect stable fixed point. With this, we introduce the error measure

$$e_\Psi = \frac{2}{\alpha_0^2} \iint_\Psi \mathrm{d}A,$$

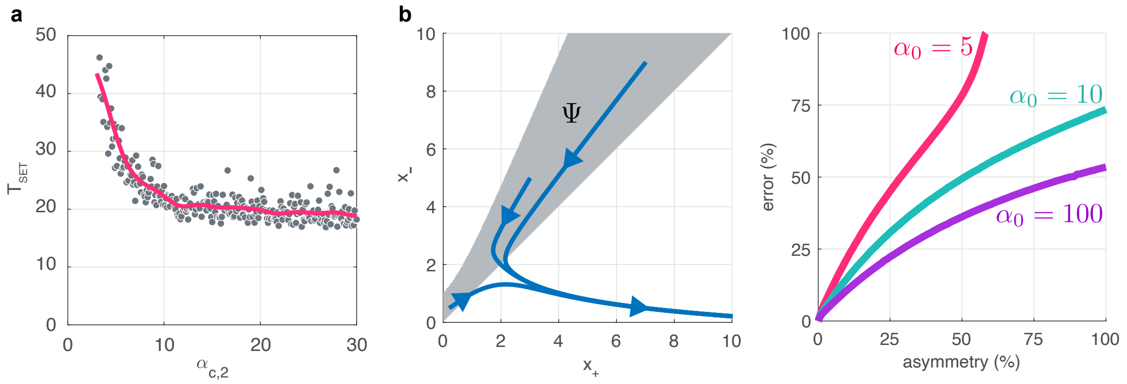

Supplementary Fig. 3. **Performance of the comparator module.** **a** The settling time T_{SET} in phase 2 is computed numerically: gray circles represent simulation data considering 10^4 independently selected initial points for each value of $\alpha_{c,2}$, whereas the red curve denotes the trend using Gaussian smoothing. Data is displayed for $\epsilon_c = 1$ and $\delta = 0.05\alpha_{c,2}$. **b** Without resource sequestration ($\beta_{x_+} = \beta_{x_-} = 0$), parameter asymmetry ($\alpha_{x_+} = 12.5$, $\alpha_{x_-} = 7.5$) causes significant error e_Ψ, thus trajectories converge to the "incorrect" stable fixed point. The error e_Ψ increases with parameter asymmetry δ_0.

where $\alpha_0 = \sqrt{\alpha_{x_+}\alpha_{x_-}}$. Therefore, e_Ψ characterizes the fraction of the half-square $[0, \alpha_0]^2$ with incorrect final state (e.g., $e_\Psi = 0$ if there is no error and $e_\Psi \to 1$ as the toggle gets increasingly more unbalanced).

As illustrated in Supplementary Fig. 3b, greater values of α_0 yield lower error e_Ψ for a given asymmetry $\delta_0 = 1 - \alpha_{x_+}/\alpha_{x_-}$. This error can be mitigated, for instance, by leveraging competition for shared cellular resources (*19*). In particular, once the scarcity of these resources is accounted for, the dynamics of the toggle switch are given by

$$\frac{\mathrm{d}x_+}{\mathrm{d}t} = \frac{\frac{\alpha_{x_+}}{1+x_-^2}}{1 + \frac{\beta_{x_+}}{1+x_-^2} + \frac{\beta_{x_-}}{1+x_+^2}} - x_+, \qquad \frac{\mathrm{d}x_-}{\mathrm{d}t} = \frac{\frac{\alpha_{x_-}}{1+x_+^2}}{1 + \frac{\beta_{x_+}}{1+x_-^2} + \frac{\beta_{x_-}}{1+x_+^2}} - x_-. \tag{7}$$

Via the optimal choice of the parameters β_{x_+} and β_{x_-} representing resource usage, the region Ψ can be significantly reduced. In particular, for $\alpha_0 > 15$ the error can be almost completely eliminated (*19*).

1.4 Logic module

The standard model of a logic AND gate with inputs A and B and output C is given by

$$\dot{C} = \alpha_C \frac{A^{n_A}}{K_A^{n_A} + A^{n_A}} \frac{B^{n_B}}{K_B^{n_B} + B^{n_B}} - \gamma_C C,$$

where K_A and K_B are the dissociation constants of A and B with Hill coefficients n_A and n_B, respectively, and α_C and γ_C are production and decay rate constants of C, respectively. Similarly, the dynamics of the logic OR gate with inputs A and B and output C are given by

$$\dot{C} = \alpha_C \left[\frac{A^{n_A}}{K_A^{n_A} + A^{n_A}} + \frac{B^{n_B}}{K_B^{n_B} + B^{n_B}} \right] - \gamma_C C.$$

Consider first the AND gates in Fig. 4 with output species Q_{++}, Q_{--}, Q_{+-}, and Q_{-+}. To simplify notation, we assume that these species have identical production and decay rate constants, denoted by α_l and γ_l, respectively. Furthermore, we assume that the input species (i.e., x_-, x_+, y_-, y_+) all act as dimers and they have identical dissociation constants denoted by $K_\wedge$. With this, we define

$$H_\wedge(A, B) = \frac{A^2}{K_\wedge^2 + A^2} \frac{B^2}{K_\wedge^2 + B^2},$$

yielding the dynamics

$$\begin{aligned} \dot{Q}_{++} &= \alpha_l H_\wedge(x_+, y_+) - \gamma_l Q_{++}, & \dot{Q}_{--} &= \alpha_l H_\wedge(x_-, y_-) - \gamma_l Q_{--}, \\ \dot{Q}_{+-} &= \alpha_l H_\wedge(x_+, y_-) - \gamma_l Q_{+-}, & \dot{Q}_{-+} &= \alpha_l H_\wedge(x_-, y_+) - \gamma_l Q_{-+}, \end{aligned}$$

so that with $q = Q\gamma_l/\alpha_l$ for $q \in \{q_{++}, q_{+-}, q_{-+}, q_{--}\}$ and $Q \in \{Q_{++}, Q_{+-}, Q_{-+}, Q_{--}\}$, we obtain that

$$\begin{aligned} \epsilon_l \dot{q}_{++} &= H_\wedge(x_+, y_+) - q_{++}, & \epsilon_l \dot{q}_{+-} &= H_\wedge(x_+, y_-) - q_{+-}, \\ \epsilon_l \dot{q}_{-+} &= H_\wedge(x_-, y_+) - q_{-+}, & \epsilon_l \dot{q}_{--} &= H_\wedge(x_-, y_-) - q_{--}, \end{aligned}$$

where $\epsilon_l = 1/\gamma_l$. Hence, at the steady state we have $q_{++} = H_\wedge(x_+, y_+)$, $q_{+-} = H_\wedge(x_+, y_-)$, $q_{-+} = H_\wedge(x_-, y_+)$, and $q_{--} = H_\wedge(x_-, y_-)$.

Similarly, the dynamics of the OR gates in Fig. 4 are governed by

$$\dot{U}_1 = \alpha_l \left(\frac{Q_{++}^2}{K_Q^2 + Q_{++}^2} + \frac{Q_{--}^2}{K_Q^2 + Q_{--}^2} \right) - \gamma_l U_1,$$
$$\dot{U}_2 = \alpha_l \left(\frac{Q_{+-}^2}{K_Q^2 + Q_{+-}^2} + \frac{Q_{-+}^2}{K_Q^2 + Q_{-+}^2} \right) - \gamma_l U_2,$$

where K_Q denotes the dissociation constant of the output species of the AND gates (i.e., Q_{++}, Q_{+-}, Q_{-+}, Q_{--}), whereas α_l and γ_l are production and decay rate constants of the (output) species U_i, respectively ($i = 1, 2$). Let $K_\vee = K_Q \gamma_l / \alpha_l$ and define

$$H_\vee(A, B) = \frac{A^2}{K_\vee^2 + A^2} + \frac{B^2}{K_\vee^2 + B^2},$$

together with $u_i = U_i \gamma_l / \alpha_l$ for $i = 1, 2$, yielding $\epsilon_l \dot{u}_1 = H_\vee(q_{++}, q_{--}) - u_1$ and $\epsilon_l \dot{u}_2 =$

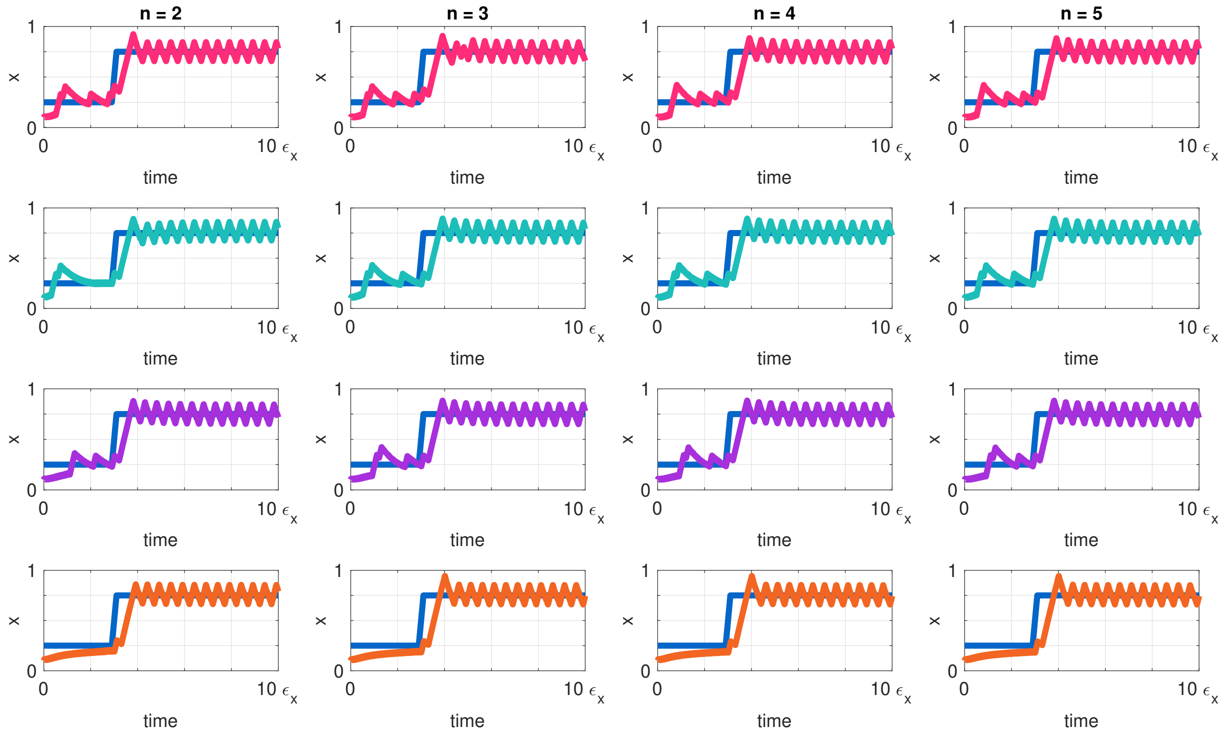

Supplementary Fig. 4. **Closed loop performance is unaffected by changes in the value of the Hill coefficient n.** Simulation parameters are identical to those in Fig. 4, except for the value of n, as indicated above the top row. Blue curve denotes the time-varying location of the optimum.

$H_\vee(q_{+-}, q_{-+}) - u_2$. Thus, at the steady state we obtain $u_1 = H_\vee(H_\wedge(x_+, y_+), H_\wedge(x_-, y_-))$ and $u_2 = H_\vee(H_\wedge(x_+, y_-), H_\wedge(x_-, y_+))$.

Data presented in Supplementary Fig. 4 highlight that the value of the Hill coefficient is not a crucial design parameter, as closed loop performance is robust to changes in it. Supplementary Fig. 5 further shows that the performance of the optimizer is unaffected by the value of ϵ_l as long as it is smaller than ϵ_d, i.e., the dynamics of the logic gates are faster than those of the delay module. Past this point, the performance quickly deteriorates.

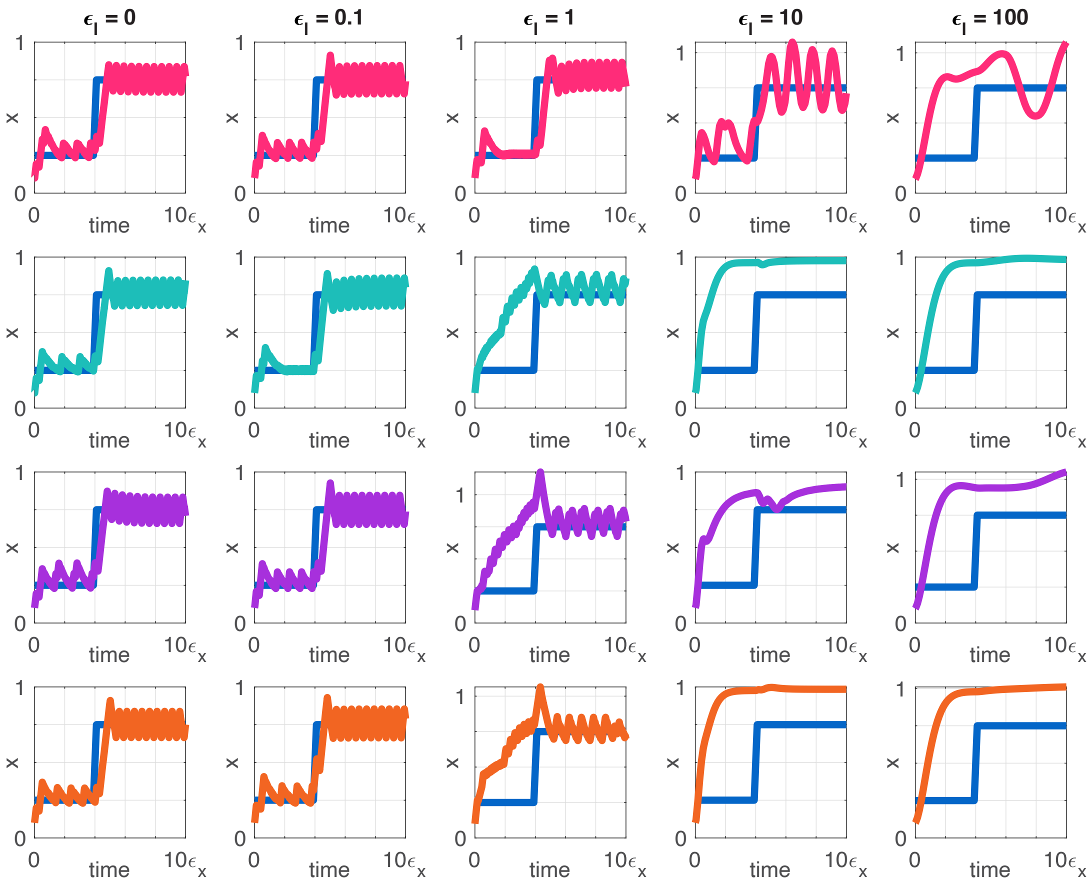

Supplementary Fig. 5. **Closed loop performance is not affected by the value of ϵ_l as long as the dynamics of the logic module are sufficiently fast (i.e., $\epsilon_l \ll \epsilon_d$).** Simulation parameters are identical to those in Fig. 4, except for ϵ_l: from left to right, in subsequent columns we have $\epsilon_l = 0$, $\epsilon_l = \epsilon_c$, $\epsilon_l = \epsilon_d$, and $\epsilon_l = \epsilon_x$. Blue curve denotes the time-varying location of the optimum.

1.5 Closed loop performance of the simplified optimizer

In Supplementary Fig. 6a, we demonstrate that the optimizer ensures (near) optimal performance independent of the location of the optimum and of the initial value of x. However, the rate of convergence does depend on these factors (Supplementary Fig. 6a).

The proposed optimizer operates in discrete time steps of length τ (the period of the comparator), where the value at the beginning of the kth step is x_k (and similarly y_k for y). Considering this simplified model of the optimizer, the differences $\Delta x_k = x_k - x_{k-1}$ and $\Delta y_k = y_k - y_{k-1}$ determine the value of the control signals. As detailed in the main text, we have $(u_1, u_2) = (1, 0)$

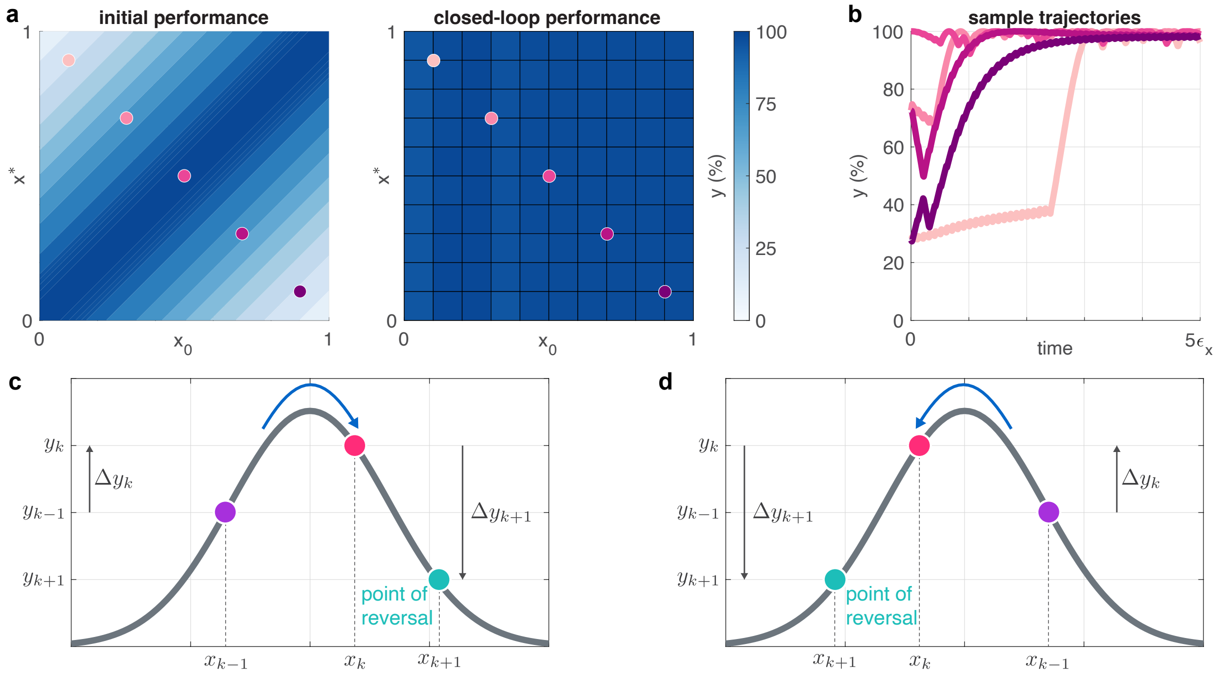

Supplementary Fig. 6. **Closed loop performance of the simplified optimizer.** **a** Initial performance starting from $x(0) = x_0$ with the optimum located at x^* and closed loop performance corresponding to (x_0, x^*) at the center of each cell, averaged over the time interval $[4\epsilon_x, 5\epsilon_x]$ considering three independent simulations. The objective function takes the form of (2), and the simulation parameters are $\sigma = 0.5$, $\epsilon_x = 100$, $\epsilon_d = 10$, $\epsilon_y = 1$, $\epsilon_c = \epsilon_l = 0.1$, $\alpha_d = \alpha_{c,1} = 1$ $\alpha_{c,2} = 100$, $K_\wedge = 1$, $K_\vee = 0.01$, $n = 2$, $\tau = 10$, and the duty cycle of the oscillator is 10%. **b** Sample trajectories correspond to the dots of the same color in panel a. **c** The optimum is crossed while increasing x. **d** The optimum is crossed while decreasing x.

over the kth step if $\Delta x_k \Delta y_k > 0$ and $(u_1, u_2) = (0, 1)$ otherwise. Therefore, in the former and latter cases, the closed loop dynamics of x are given by $\epsilon_x \dot{x} = 1$ and $\epsilon_x \dot{x} = -x$, respectively, leading to $x_{k_1} = x_k + \tau/\epsilon_x$ and $x_{k_1} = x^* e^{-\tau/\epsilon_x}$, respectively, where the duration of the step is τ. With this, assume that the optimum is crossed during the kth step, either when increasing x (Supplementary Fig. 6c) or when decreasing it (Supplementary Fig. 6d). Thus, during this step (purple-red transition in Supplementary Fig. 6cd), the optimum is first approached and then crossed, after which changes in x lead away from the optimum until the step is concluded. Subsequently, reversal towards the optimum happens when the value of y starts to decrease. If $\Delta y_k < 0$ then this happens at x_k (red in Supplementary Fig. 6cd), otherwise at x_{k+1} (green in Supplementary Fig. 6cd). Therefore, oscillations around the optimum x^* are confined within $[x^* e^{-2\tau/\epsilon_x}, x^* + 2\tau/\epsilon_x]$, thus the amplitude increases with τ/ϵ_x.

2 Implementation based on existing synthetic biology parts and components

Here, we first present the detailed mathematical model of the whole integrated system featured in Fig. 6, then review the typical range of model parameters. Based on this, we outline the realistic parameter values underpinning the simulation data presented in Fig. 7–8. Finally, we demonstrate that closed loop performance is robust to variations in parameter values. In what follows, production and degradation of RNA r and protein p in cells growing at rate μ are modeled as one-step processes given by

$$\dot{r} = \alpha - (\mu + \delta)\, r, \qquad \dot{p} = \beta - (\mu + \lambda)\, p, \tag{8}$$

where α and δ denote the RNA production and degradation rate constants, respectively, and β and λ denote the protein production and degradation rate constants, respectively. RNA-DNA and protein-DNA dissociation constants are denoted by k and K, respectively.

2.1 Dynamics of the integrated system presented in Fig. 6

To simplify notation, we write $F(x)$ instead of $F(x, \theta_y)$ in what follows. We further assume that basal expression rates are negligible, which can be ensured via stringent multi-level control (*20*).

Delay Tracking of x and y via the proteins x_d and y_d occur according to

$$\dot{x}_d = \beta_{x_d} \frac{x}{x + K_x} - (\mu + \lambda_d)\, x_d, \qquad \dot{y}_d = \beta_{y_d} \frac{y}{y + K_y} - (\mu + \lambda_d)\, y_d. \tag{9}$$

Oscillator The repressilator dynamics with proteins o_1, o_2, and o_3 and the activator a co-expressed with the repressor $r = o_3$ are given by

$$\begin{aligned} \dot{o}_1 =& \beta_o \frac{1}{1 + (o_3/K_o)^{n_{o_3}}} - (\mu + \lambda_o)\, o_1, \qquad \dot{o}_2 = \beta_o \frac{1}{1 + (o_1/K_o)^{n_{o_1}}} - (\mu + \lambda_o)\, o_2, \\ \dot{o}_3 =& \beta_o \frac{1}{1 + (o_2/K_o)^{n_{o_2}}} - (\mu + \lambda_o)\, o_3, \qquad \dot{a} = \beta_o' \frac{1}{1 + (o_2/K_o)^{n_{o_2}}} - (\mu + \lambda_o)\, a. \end{aligned} \tag{10}$$

Unless otherwise stated, we assume that $\beta_o = \beta_o'$.

Toggle switches The dynamics of the gRNAs are given by

$$\begin{aligned} \dot{x}_- =& \alpha_x \frac{K_c^{n_r}}{r^{n_r} + K_c^{n_r}} \frac{x_d}{x_d + \bar{K}_x} + \bar{\alpha}_x \frac{a^{n_a}}{a^{n_a} + K_c^{n_a}} \frac{\bar{k}_x^2}{x_+^2 + \bar{k}_x^2} - (\mu + \delta_c)\, x_-, \\ \dot{x}_+ =& \alpha_x \frac{K_c^{n_r}}{r^{n_r} + K_c^{n_r}} \frac{x}{x + \bar{K}_x} + \bar{\alpha}_x \frac{a^{n_a}}{a^{n_a} + K_c^{n_a}} \frac{\bar{k}_x^2}{x_-^2 + \bar{k}_x^2} - (\mu + \delta_c)\, x_+, \end{aligned} \tag{11}$$

$$\begin{aligned} \dot{y}_- =& \alpha_y \frac{K_c^{n_r}}{r^{n_r} + K_c^{n_r}} \frac{y_d}{y_d + \bar{K}_y} + \bar{\alpha}_y \frac{a^{n_a}}{a^{n_a} + K_c^{n_a}} \frac{\bar{k}_y^2}{y_+^2 + \bar{k}_y^2} - (\mu + \delta_c)\, y_-, \\ \dot{y}_+ =& \alpha_y \frac{K_c^{n_r}}{r^{n_r} + K_c^{n_r}} \frac{y}{y + \bar{K}_y} + \bar{\alpha}_y \frac{a^{n_a}}{a^{n_a} + K_c^{n_a}} \frac{\bar{k}_y^2}{y_-^2 + \bar{k}_y^2} - (\mu + \delta_c)\, y_+, \end{aligned} \tag{12}$$

with $n_a = n_r = n_{o_3}$ and $K_c = 10K_o$. The dynamics of the STARs are similarly given by

$$\begin{aligned} \dot{\bar{x}}_- =& \bar{\alpha}_x \frac{a^{n_a}}{a^{n_a} + K_c^{n_a}} \frac{\bar{k}_x^2}{x_+^2 + \bar{k}_x^2} - (\mu + \delta_c)\, \bar{x}_-, \\ \dot{\bar{x}}_+ =& \bar{\alpha}_x \frac{a^{n_a}}{a^{n_a} + K_c^{n_a}} \frac{\bar{k}_x^2}{x_-^2 + \bar{k}_x^2} - (\mu + \delta_c)\, \bar{x}_+, \end{aligned} \tag{13}$$

$$\dot{\bar{y}}_- = \bar{\alpha}_y \frac{a^{n_a}}{a^{n_a} + K_c^{n_a}} \frac{\bar{k}_y^2}{y_+^2 + \bar{k}_y^2} - (\mu + \delta_c)\, \bar{y}_-,$$
$$\dot{\bar{y}}_+ = \bar{\alpha}_y \frac{a^{n_a}}{a^{n_a} + K_c^{n_a}} \frac{\bar{k}_y^2}{y_-^2 + \bar{k}_y^2} - (\mu + \delta_c)\, \bar{y}_+. \tag{14}$$

Logic gates The dynamics of the AND gates are given by

$$\dot{q}_{++} = \alpha_q \frac{\bar{x}_+}{\bar{x}_+ + k'_x} \frac{\bar{y}_+}{\bar{y}_+ + k'_y} - (\mu + \delta_q)\, q_{++},$$
$$\dot{q}_{--} = \alpha_q \frac{\bar{x}_-}{\bar{x}_- + k'_x} \frac{\bar{y}_-}{\bar{y}_- + k'_y} - (\mu + \delta_q)\, q_{--},$$
$$\dot{q}_{+-} = \alpha_q \frac{\bar{x}_+}{\bar{x}_+ + k'_x} \frac{\bar{y}_-}{\bar{y}_- + k'_y} - (\mu + \delta_q)\, q_{+-},$$
$$\dot{q}_{-+} = \alpha_q \frac{\bar{x}_-}{\bar{x}_- + k'_x} \frac{\bar{y}_+}{\bar{y}_+ + k'_y} - (\mu + \delta_q)\, q_{-+}. \tag{15}$$

Similarly, the outputs of the OR gates evolve according to

$$\dot{u}_1 = \beta_u \left(\frac{q_{++}}{q_{++} + k_q} + \frac{q_{--}}{q_{--} + k_q} \right) - (\mu + \lambda_u)\, u_1,$$
$$\dot{u}_2 = \beta_u \left(\frac{q_{+-}}{q_{+-} + k_q} + \frac{q_{-+}}{q_{-+} + k_q} \right) - (\mu + \lambda_u)\, u_2. \tag{16}$$

Regulator The dynamics of the regulator x and the protease v are given by

$$\dot{x} = \beta_x \frac{x}{x + K_x} + \bar{\beta}_x \frac{u_1^2}{u_1^2 + K_{u_1}^2} - \left[\mu + \lambda_x \left(1 + \nu v\right)\right] x,$$
$$\dot{v} = \beta_v \frac{u_2}{u_2 + K_{u_2}} - (\mu + \lambda_v)\, v, \tag{17}$$

where the parameter ν characterizes how rapidly the protease v degrades x. Weak self-activation of x is introduced so that degradation of x is primarily driven by controlled decay via v, which is ensured when $\beta_x \approx K_x(\mu + \lambda_x)$.

Reporter With $F(x)$ characterizing the direct impact of x on y (more complex cases are considered in Fig. 7–8), the dynamics of y are given by

$$\dot{y} = F(x) - (\mu + \lambda_y)\, y. \tag{18}$$

Unless otherwise stated, $F(x)$ takes the form of (2) with $F_0 = 100$ nM h^{-1} and $\sigma = 20$ nM.

Supplementary Table 1. Parameters of the optimizer used across all application examples.

	Parameter	Value	Unit
growth rate	μ	1	h^{-1}
RNA degradation rate constant	δ_c	20	h^{-1}
	δ_q	6	h^{-1}
protein degradation rate constant	λ_x	0.2	h^{-1}
	λ_y	1	h^{-1}
	λ_v	4	h^{-1}
	λ_d	4	h^{-1}
	λ_u	6	h^{-1}
	λ_o	6	h^{-1}
RNA production rate constant	α_x	21	μM h^{-1}
	α_y	2.1	μM h^{-1}
	$\bar{\alpha}_x$	21	μM h^{-1}
	$\bar{\alpha}_y$	10.5	μM h^{-1}
	α_q	0.7	μM h^{-1}
protein production rate constant	β_x	6.1	μM h^{-1}
	$\bar{\beta}_x$	0.12	μM h^{-1}
	β_v	2	μM h^{-1}
	β_{x_d}	25	μM h^{-1}
	β_{y_d}	25	μM h^{-1}
	β_u	3.5	μM h^{-1}
	β_o	20	μM h^{-1}
RNA-DNA dissociation constant	$\bar{k}_x$	100	nM
	$\bar{k}_y$	10	nM
	k'_x	1	μM
	k'_y	1	μM
	k_q	500	nM
protein-DNA dissociation constant	K_o	1	nM
	K_x	5	μM
	K_y	5	μM
	K_{u_1}	40	nM
	K_{u_2}	200	nM
	$\bar{K}_x$	1	μM
	$\bar{K}_y$	100	nM
Hill coefficient	n_{o_1}	2	-
	n_{o_2}	2	-
	n_{o_3}	4	-

2.2 Model parameters

Parameter values of the genetic optimizer are fixed across all simulations, see Supplementary Table 1. These values fall within the typical ranges in *E. coli*, as we detail below, and can be modulated by relying on standard synthetic biology tools in a straightforward fashion by adjusting transcriptional, translational, degradation, and dissociation rate constants (*20–23*).

Bacterial growth Depending on the strain and media composition (among other factors), doubling time of *E. coli* usually ranges between 20 min and 2 h (*24–31*). As a result, growth rate typically varies between $\mu = 0.35$ h^{-1} and $\mu = 2$ h^{-1}.

Cell volume The concentration of a single molecule during exponential growth corresponds to about 1 nM since the typical volume of *E. coli* cells is approximately 10^{-18} m^3 (*32, 33*).

RNA degradation RNA half-life in *E. coli* displays considerable variation ranging from 40 seconds to 20 minutes (*34–36*). While for most genes it is typically between 2–10 minutes (*37*), it can be even lower for short monocistronic mRNAs (*38–42*). Genome-wide RNA-seq data in *E. coli* further estimates the median lifetime to be 2.8 min, corresponding to 2 min half-life (*43, 44*), somewhat shorter than previous genome-wide microarray measurements (*45, 46*), most likely due to the correction for residual RNAP activity employed in (*43, 44*). These measurements confirm prior estimates across a variety of growth rates ranging from 0.6 h^{-1} to 3 h^{-1} (*28*) and characterizations across different media compositions (*47, 48*). Based on these, we assume that RNA degradation rate δ in (8) varies between 4 h^{-1} and 30 h^{-1}.

RNA production Transcription initation in *E. coli* takes approximately 1–3 s (*47, 49, 50*), whereas elongation occurs at a rate of 40–50 nucleotides per second (*49, 51*). The average length of proteins is approximately 1000 nucleotides (*52*), hence typical genes are transcribed

at a rate of approximately $100\,\mathrm{nM\,h^{-1}}$. This estimate is further confirmed by genome-wide transcriptome analysis using RNA-seq data (*43, 44*). For short RNA products, however, the effective production rate can be up to 20 times greater due to rapid elongation, thus for the gRNA and STAR molecules in our circuit we consider α to be typically 0.1–2 μM h^{-1} (which can be increased substantially, for instance, by tuning the plasmid copy number (*53*)).

RNA-DNA binding strength The dissociation constant K of the gRNA-Cas9 complex with the matching region of the DNA is reported to be 1–10 nM (*54, 55*) with a half-life of 5 min (*54*). The value of K can be easily increased and the half-life of the binding decreased via the introduction of mismatches, and by varying their number, position and distribution (*56–59*), rendering the interaction up to 1000-fold weaker (*60*). Alternatively, weaker binding can be achieved by relying on truncated gRNAs (*61, 62*), modified RNA secondary structures (*63*), and by modifying the endonuclease to weaken electrostatic interactions with the DNA backbone (*64, 65*), strategies that are used for increased specificity of CRISPR-based genome editing (*66*).

Protein degradation Proteins can remain stable over several generations with apparent half-lives of 5–20 hours, or can be highly unstable with half-lives as short as 1–2 minutes (*67*). As a result, protein degradation rate can be efficiently tuned via degradation tags: for instance, while GFP half-life in *E. coli* is reported to be approximately 10 hours, variants of the *E. coli* ssrA tag can decrease it over 100-fold to 2–6 minutes (*23, 68*), and similar rates can be obtained when these tags are fused to LacI (*69*). Additionally, considering the *E. coli* Lon protease and a typical gene comprising 1000 nucleotides (*52*), relying on the β20 tag (the 20-residue β-galactosidase sequence) yields a half-life of approximately 4 min, whereas with the sul20C tag (the C-terminal 20 residues of SulA) it is further reduced to about 1 min (*70, 71*). As a result, protein degradation rate is estimated to range between $\lambda \approx 0\,\mathrm{h^{-1}}$ for highly stable proteins to $\lambda \approx 30\,\mathrm{h^{-1}}$ for rapidly degraded variants with strong degradation tags.

Protein production The abundance of typical endogenous *E. coli* proteins is approximately 10^2–10^3 copies per cell, though the most abundant proteins may have molecular counts 10^3 times greater (*37, 72–77*). Considering a typical endogenous protein and depending on the degradation rate, from (8) we estimate that the production rate thus varies between $\beta \approx 0.1$–10 μM h^{-1} (which can be increased substantially, e.g., by tuning the plasmid copy number (*53*)).

Protein-DNA binding strength The typical range for the dissociation constant K for some of the most common transcription factors in *E. coli* ranges between 0.1 nM and 1 μM (*78–80*). Mutations can alter K by two-three orders of magnitude (*21, 81–83*), especially to weaken it by decreasing the match between a transcription factor and its cognate binding site.

Oscillator parameters Given its widespread use (*84–88*), we included the repressilator to serve as the oscillator in the comparator module, and selected its parameters to obtain a period length of approximately 2 h (Supplementary Fig. 7), well within the range of already existing experimental realizations (*89–91*). We consider the standard implementation of the repressilator (*89–91*) relying on cI from λ phage (o_1), LacI (o_2), and TetR (o_3) harbored in a plasmid equipped with the pSC101 origin of replication with approximately 4 copies per cell (*92–97*).

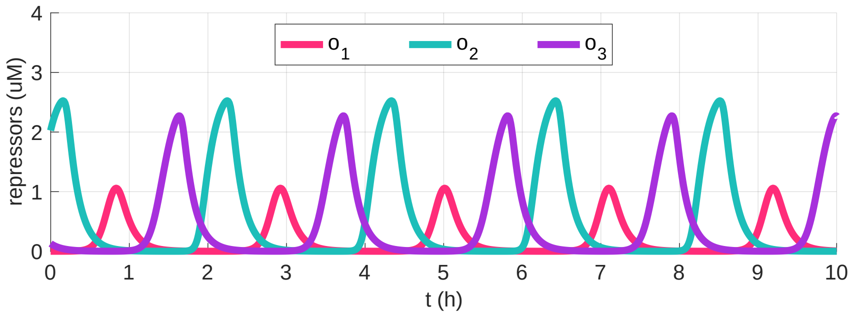

Supplementary Fig. 7. **Periodic signal generated by the phase selector oscillator.** The dynamics of the phase selector are given by (10) with $\beta_o = 20$ μM h^{-1}, $K_o = 1$ nM, $\lambda_o = 6$ h^{-1}, $n_{o_1} = 2$, $n_{o_2} = 2$, and $n_{o_3} = 4$.

As a result, the corresponding Hill coefficients are $n_{o_1} = 2$, $n_{o_2} = 2$, and $n_{o_3} = n_r = 4$ (*98*), together with the estimated production rate constant $\beta_o = 20\ \mu\text{M h}^{-1}$ and degradation rate constant $\lambda_o = 6\ \text{h}^{-1}$. TetR can serve as the repressor r, whereas the activator co-expressed with it could be VP64 (*99–101*), a tetrameric TF ($n_a = 4$) to ensure rapid transitions between the on and off states. Closed loop performance is robust to changes in the period length (Supplementary Fig. 8). Unsurprisingly, as the "step-size" decreases with shorter periods, closed loop performance improves as λ_o increases. Finally, closed loop performance is also insensitive to differences between the production rate constant of a and r (Supplementary Fig. 9).

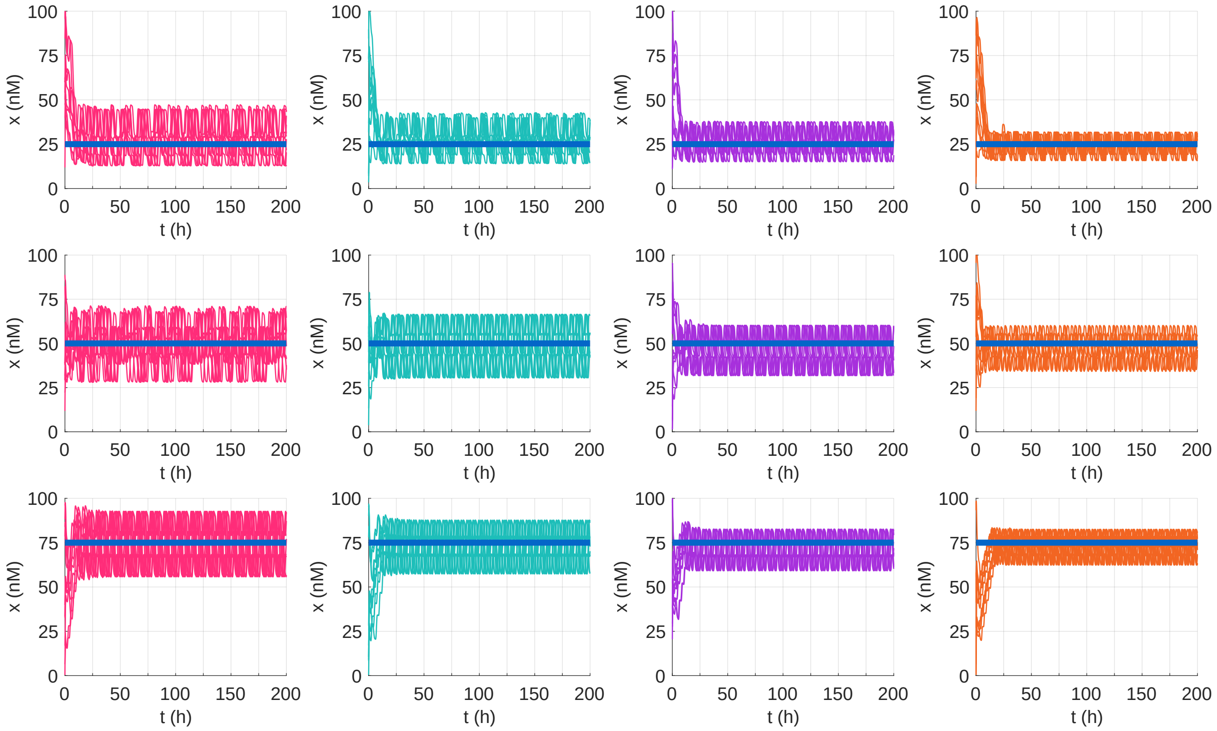

Supplementary Fig. 8. **Impact of cycle length on closed loop performance.** Blue lines denote the location of the optimum, simulation data correspond to 10 independent runs with random initial conditions. Parameters of the optimizer module are taken from Supplementary Table 1. Degradation rate constant of the repressilator proteins are $\delta_o = 4\ \text{h}^{-1}$ (red), $\delta_o = 4.6\ \text{h}^{-1}$ (green), $\delta_o = 5.3\ \text{h}^{-1}$ (purple), and $\delta_o = 6\ \text{h}^{-1}$ (orange), corresponding to cycle lengths of $T = 3$ h, $T = 2.7$ h, $T = 2.3$ h, and $T = 2$ h, respectively. As the growth rate $\mu = 1\ \text{h}^{-1}$ results in approximately 40 min generation time, these correspond to 4.5, 4.0, 3.5, and 3.0 generations.

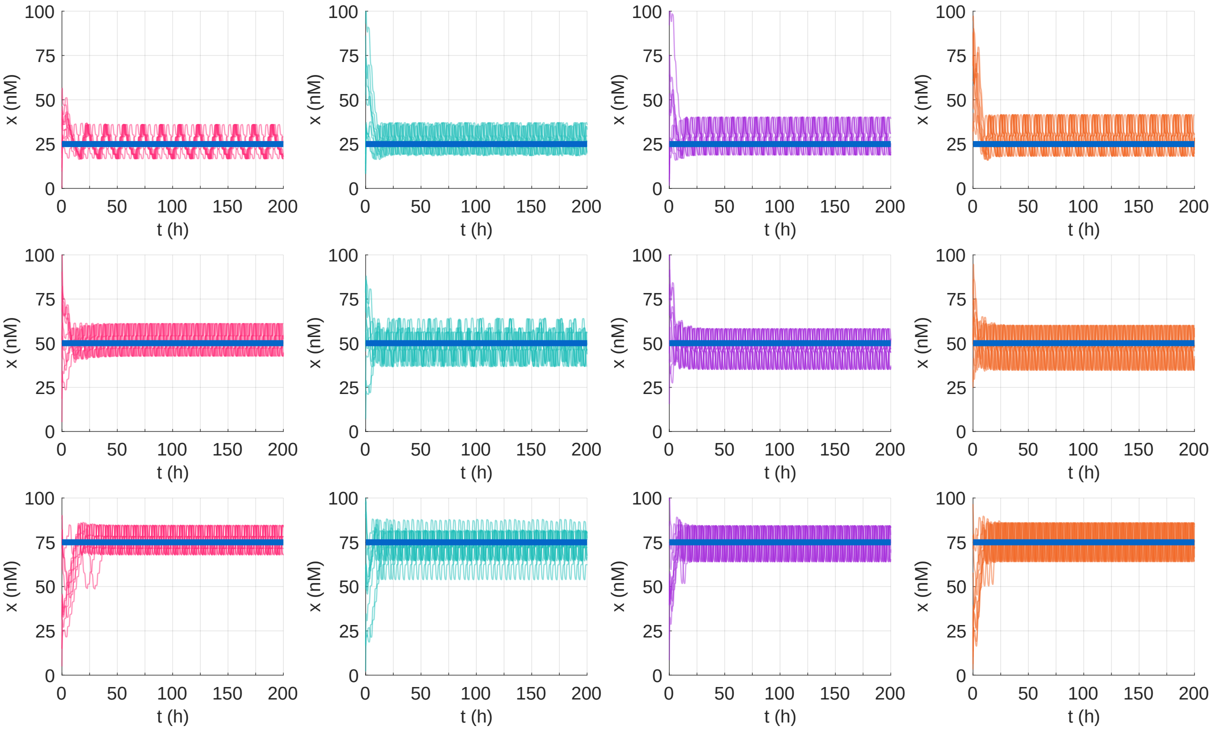

Supplementary Fig. 9. **Impact of uneven production of the activator and the repressor in the phase selector oscillator on closed loop performance.** Blue lines denote the location of the optimum, simulation data correspond to 10 independent runs with random initial conditions. Parameters of the optimizer module are taken from Supplementary Table 1, together with $\beta_o' = \beta_o/2$ (red), $\beta_o' = \beta_o$ (green), $\beta_o' = 2\beta_o$ (purple), $\beta_o' = 5\beta_o$ (orange).

Regulator parameters We selected $\nu = 0.05$ nM^{-1} to ensure that $\max(\nu v) \approx 1$, that is, the protease v doubles the degradation rate of x, well within the 30-fold range that can be achieved using controlled protein degradation (*102*). Closed loop performance is robust to changes in ν as its value can be doubled without negligible impact (Supplementary Fig. 10). Similarly, while ideally the value of β_x is chosen such that $\beta_x \approx K_x(\mu + \lambda_x)$ to ensure that the expression and removal of x is primarily due to regulation via u_1 and u_2, a precise match is not a requirement for correct functioning (Supplementary Fig. 10).

Objective function parameters Data in Supplementary Fig. 11–Supplementary Fig. 13 highlight that closed loop performance is robust to changes in the parameters of the objective func-

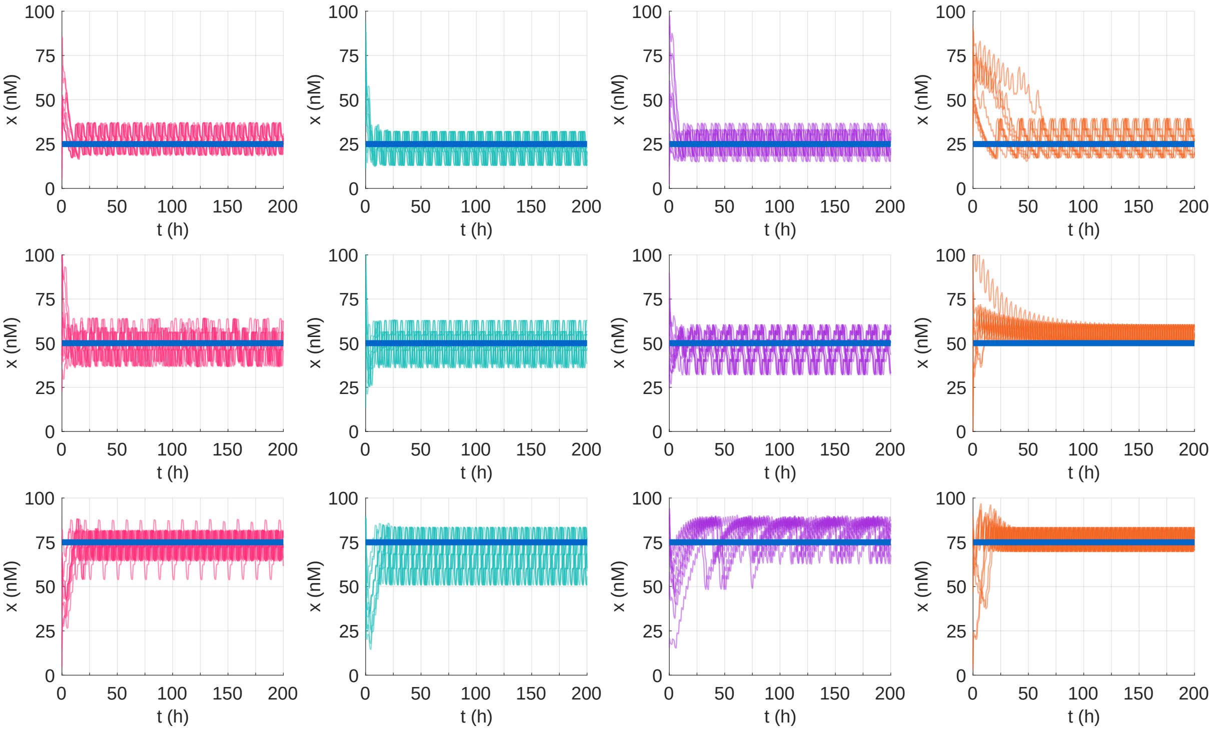

Supplementary Fig. 10. **Impact of changes in the regulator parameters on closed loop performance.** Blue lines denote the location of the optimum, simulation data correspond to 10 independent runs with random initial conditions. Parameters of the optimizer module are taken from Supplementary Table 1 together with $\nu_0 = 0.05$ nM^{-1} h^{-1} and $\beta_{x,0} = 6.1$ μM h^{-1}, such that $(\nu, \beta_x) = (\nu_0, \beta_{x,0})$ for red, $(\nu, \beta_x) = (2\nu_0, \beta_{x,0})$ for green, $(\nu, \beta_x) = (\nu_0, 0.97\beta_{x,0})$ for purple, and $(\nu, \beta_x) = (\nu_0, 1.03\beta_{x,0})$ for orange.

tion (2). In these examples we considered the optimal concentration x^* to fall below 100 nM due to the fact that the total protein abundance may far exceed the concentration of its free monomeric form. To illustrate this, consider first RNA polymerase (RNAP): while the number of free RNAP molecules per cell can be as low as 100, it only represents less than 10% of the total pool (*27*). The effect can be even more dramatic when proteins form tight multimers. For instance, LacI L first forms the homodimer L_2, then these dimerize to form L_4 according to

$$L + L \underset{\kappa_2^-}{\overset{\kappa_2^+}{\rightleftharpoons}} L_2, \qquad L_2 + L_2 \underset{\kappa_4^-}{\overset{\kappa_4^+}{\rightleftharpoons}} L_4,$$

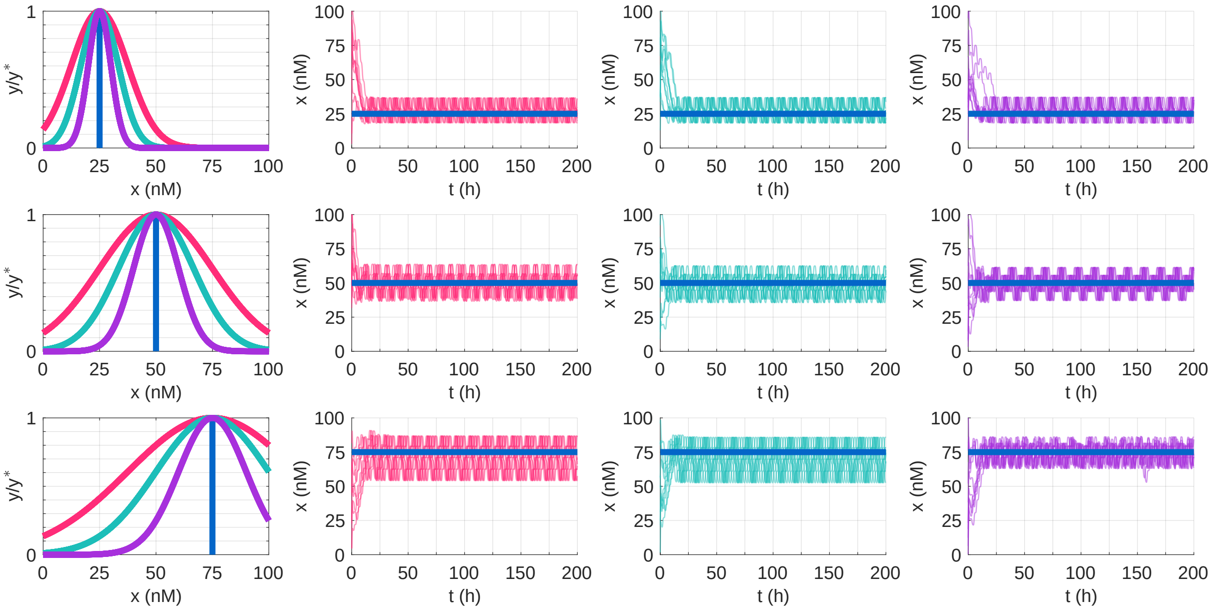

Supplementary Fig. 11. **Impact of objective function parameters on closed loop performance (low F_0).** Blue lines denote the location of the optimum. Parameters of the optimizer module are taken from Supplementary Table 1. The objective function takes the form specified in (2) with $\sigma = x^*/2$ (red), $\sigma = x^*/3$ (green), and $\sigma = x^*/5$ (purple). In all plots $F_0 = 50$ nM h^{-1}. The shape of the objective function does not depend on the value of F_0, only on σ and x^*.

so that at the equilibrium we have $L_2 = L^2/\kappa_2$ and $L_4 = L_2^2/\kappa_4$ with $\kappa_2 = \kappa_2^-/\kappa_2^+$ and $\kappa_4 = \kappa_4^-/\kappa_4^+$. Due to its tight multimerization, the free concentration of monomeric LacI L is much lower than its total concentration $L_T = L + L_2 + L_4$ as the overwhelming majority exists in multimeric forms: e.g., less than 0.1% when $L_T > 10$ μM (*103*). Considering the above two examples, we estimate that the free monomeric form of a protein represents approximately 0.1–10% of its total concentration. We thus expect that the optimum (free monomeric form) to fall below 100 nM when relying on common plasmids used in synthetic biology (e.g., pSC101, p15A) considering the typical concentration of proteins discussed above based on (*37, 72–77*).

In summary, the parameter values in Supplementary Table 1 that are kept fixed for all application examples featured in Fig. 7–8 and also here are biologically feasible considering the typical ranges in *E. coli*. Regarding the timescales, we picked the typical growth rate of $\mu = 1$ h^{-1},

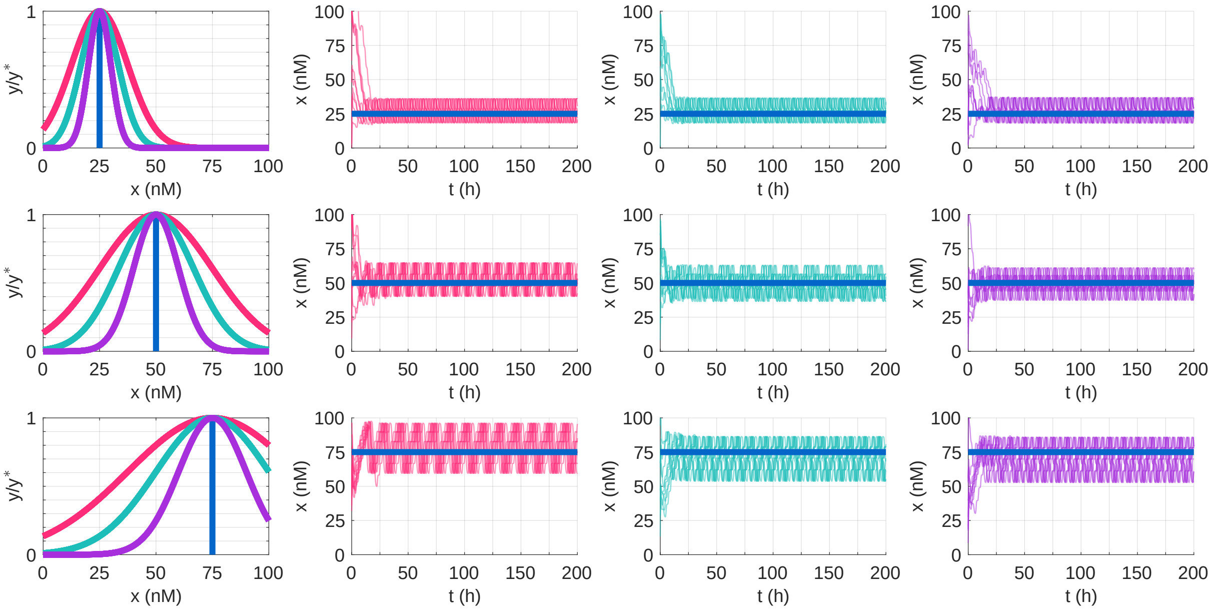

Supplementary Fig. 12. **Impact of objective function parameters on closed loop performance (medium F_0).** Blue lines denote the location of the optimum. Parameters of the optimizer module are taken from Supplementary Table 1. The objective function takes the form specified in (2) with $\sigma = x^*/2$ (red), $\sigma = x^*/3$ (green), and $\sigma = x^*/5$ (purple). In all plots $F_0 = 100$ nM h^{-1}. The shape of the objective function does not depend on the value of F_0, only on σ and x^*.

alongside with RNA degradation rate constant in the range 6–20 h^{-1}, and protein degradation rate constant in the range 0.2–6 h^{-1}. This timescale difference is typical between RNA-based and protein-based circuits: e.g., while CRISPRi-based activation/repression suffers from approximately 1–2 h delay when in addition to the gRNA the dCas9 enzyme also needs to be synthesized or degraded, the response is almost instantaneous when only the gRNA needs to be expressed or removed (*104–106*). This is in accordance with the fact while a single dCas9 enyzyme requires considerable time to find the correct target sequence, rapid overall response can be ensured by increasing dCas9 concentration (*107*) without impacting growth rate (*108*). Finally, in the implementation outlined in Fig. 6 we selected the repressilator to serve as an oscillator and chose its parameters to obtain a cycle length of 2 h, well within the range of synthetic oscillators with typical periods spanning from 13 min (*109*) to approximately 10 h (*90*).

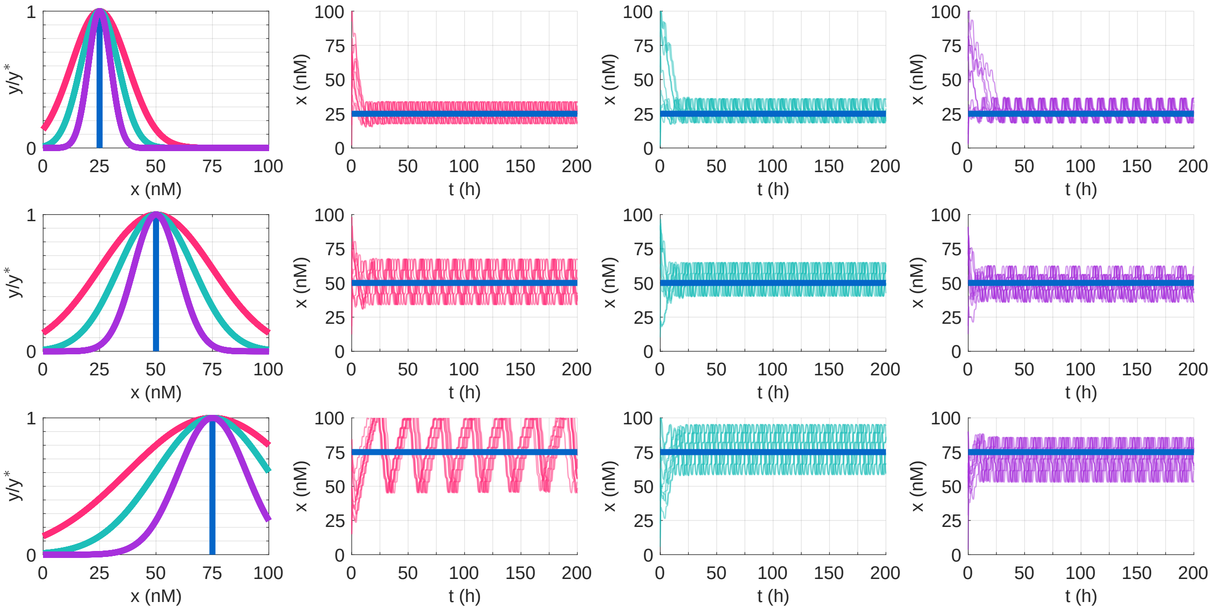

Supplementary Fig. 13. **Impact of objective function parameters on closed loop performance (high F_0).** Blue lines denote the location of the optimum. Parameters of the optimizer module are taken from Supplementary Table 1. The objective function takes the form specified in (2) with $\sigma = x^*/2$ (red), $\sigma = x^*/3$ (green), and $\sigma = x^*/5$ (purple). In all plots $F_0 = 200$ nM h^{-1}. The shape of the objective function does not depend on the value of F_0, only on σ and x^*.

3 Application examples

Here, we detail the dynamics underpinning the examples in Fig. 7–8 together with additional simulation data. Description of the simulations and parameter values are provided in Supplementary Section 5. In case of Fig. 7cd, the modified dynamics of the optimizer are included below, otherwise the dynamics of the optimizer are provided in Supplementary Section 2.1. Parameters of the optimizer are summarized in Supplementary Table 1. Transcription factor dissociation constants of the regulated pathways are denoted by κ.

Fig. 7a The dynamics of y when directly activated by x in Fig. 7a are captured by (18). To illustrate that closed loop performance is robust to changes in the shape of the objective function, consider

$$F(x) = \beta_y(x)\frac{x^{n_x}}{x^{n_x} + \kappa_F^{n_x}}, \tag{19}$$

where n_x and κ_F denote the Hill coefficient and the dissociation constant, respectively. The production rate constant $\beta_y(x)$ may begin to decrease over a threshold θ^* as x increases, for instance, due to metabolic burden (*1,3,110,111*), yielding a non-monotonic relationship despite the activation (*112*). To model this phenomenon, we may assume that $\beta_y(x) = F_0$ if $x < \theta^*$ and $\beta_y(x) = F_0/x^{n_x}$ otherwise, capturing the negative impact of protein overexpression on y (*112*). This gives rise to the non-smooth objective function

$$F(x) = \begin{cases} F_0\dfrac{x^{n_x}}{x^{n_x} + \kappa_F^{n_x}} & \text{, if } x < \theta^*, \\ F_0\dfrac{1}{x^{n_x} + \kappa_F^{n_x}} & \text{, otherwise,} \end{cases} \tag{20}$$

so that the optimum occurs at $x^* = \theta^*$. Data in Supplementary Fig. 14 demonstrate that closed loop performance is practically identical considering the smooth objective function from (2) and its non-smooth counterpart in (20), both when x acts as a monomer ($n_x = 1$) and as a homodimer ($n_x = 2$) despite the radically different shape of the objective function.

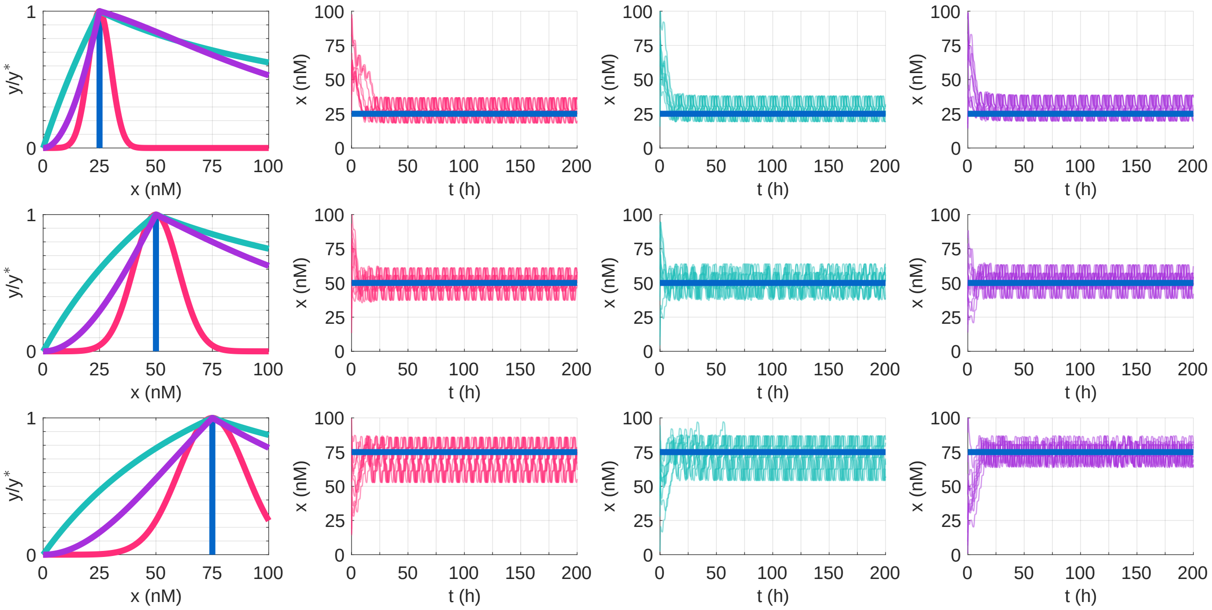

Supplementary Fig. 14. **Impact of objective function shape on closed loop performance in case of direct regulation of y via x.** Blue lines denote the location of the optimum x^*. Parameters of the optimizer module are taken from Supplementary Table 1. Red corresponds to the objective function specified in (2) with $F_0 = 100$ nM h^{-1} and $\sigma = x^*/5$. Green and purple correspond to the objective function defined in (20) when x acts as a monomer ($n_x = 1$) and as a homodimer ($n_x = 2$), respectively; in both cases $F_0 = 100$ nM h^{-1} and $\kappa_F = 100$ nM.

The dynamics of the $x = z_0 \to z_1 \to z_2 \to \cdots \to z_N \to y$ cascade in Fig. 7a are given by

$$\begin{aligned} \dot{z}_i =& \beta_z \frac{z_{i-1}}{z_{i-1} + \kappa_z} - (\mu + \lambda_z)\, z_i, \qquad i = 1, 2, \ldots, N, \\ \dot{y} =& F(z_N) - (\mu + \lambda_y) y. \end{aligned} \tag{21}$$

Considering (20), the optimum occurs at $z_N^* = \theta^*$ and $z_{i-1}^* = (\mu + \lambda_z)\, \kappa_z z_i^* / [\beta_z - (\mu + \lambda_z)\, z_i^*]$ for $i = 1, 2, \ldots, N$ at the equilibrium. Each stage introduces additional delay (decreasing with λ_z), separating the changes in x and their impact on y. As a result, closed loop performance deteriorates as N increases and as λ_z decreases (Supplementary Fig. 15).

The dynamics of the self-activated regulatory cascade $x \to \overset{\curvearrowright}{z} \to y$ in Fig. 7a are given by

$$\dot{z} = \beta_{z,1} \frac{x}{x + \kappa_x} + \beta_{z,2} \frac{z}{z + \kappa_z} - (\mu + \lambda_z)\, z, \qquad \dot{y} = F(z) - (\mu + \lambda_y)\, y. \tag{22}$$

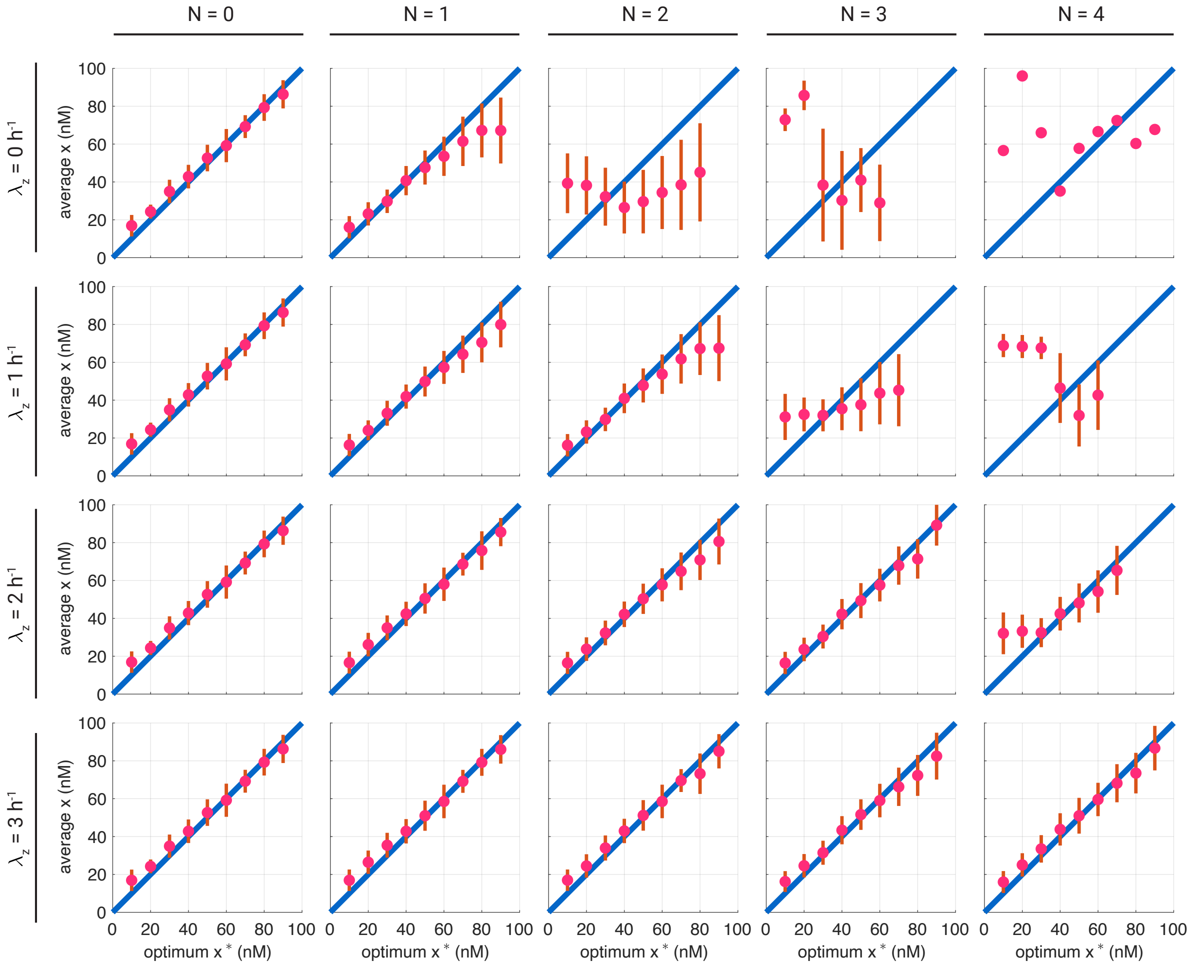

Supplementary Fig. 15. **Impact of signal propagation delay in a regulatory cascade.** Replication of the results presented in Fig. 7a when the impact of x on y is mediated via the cascade $x = z_0 \to z_1 \to z_2 \to \cdots \to z_N \to y$. Parameters of the optimizer module are taken from Supplementary Table 1, the objective function takes the form (20) with $n_x = 2$, $\kappa_F = 100$ nM, and $F_0 = 100\,\text{nM}\,\text{h}^{-1}$, together with $\lambda_y = 1\,\text{h}^{-1}$, $\beta_z = 5\,\mu\text{M}\,\text{h}^{-1}$ and $\kappa_z = 1\,\mu$M. Data was collected for $T = 200$ h. Mean and error bars denote the average of x and its standard deviation during the second half of the simulation after the initial transient has disappeared, averaged over 100 independent simulations with random initial conditions.

Fig. 7b The dynamics of the circuit with the negative feedback in Fig. 7b are given by

$$\begin{aligned}\dot{w} =& \beta_w \frac{x}{x+\kappa_x} \frac{\kappa_z}{z+\kappa_z} - (\mu + \lambda_w)\, w,\\ \dot{z} =& \beta_z \frac{w}{w+\kappa_w} - (\mu + \lambda_z)\, z,\\ \dot{y} =& F(z) - (\mu + \lambda_y)\, y.\end{aligned} \tag{23}$$

Considering (20), at the equilibrium the optimum is reached when $z^* = \theta^*$, thus

$$w^* = \frac{\kappa_w z^*}{\frac{\beta_z}{\mu+\lambda_z} - z^*}, \qquad x^* = \frac{\kappa_x w^*}{\frac{\beta_w}{\mu+\lambda_w}\frac{\kappa_z}{z^*+\kappa_z} - w^*}.$$

The dynamics of the feedforward motif in Fig. 7b can be modeled according to (*113*) as

$$\dot{z} = \beta_z \frac{\frac{x}{\kappa_x}}{1+\frac{w}{\kappa_w}} - (\mu+\lambda_z)\, z, \qquad \dot{y} = \beta_y \frac{x}{x+\kappa_x}\frac{\kappa_z}{z+\kappa_z} - (\mu+\lambda_y)\, y, \tag{24}$$

so that at the equilibrium the value of y is maximized when $x = x^*$ with

$$x^* = \kappa_x \sqrt{\kappa_z \frac{w+\kappa_w}{\kappa_w}\frac{\mu+\lambda_z}{\beta_z}}.$$

Fig. 7c The dynamics of the $x \to \tilde{y} \dashv y$ cascade are given by

$$\dot{\tilde{y}} = F(x) - (\mu+\lambda_{\tilde{y}})\, \tilde{y}, \qquad \dot{y} = \beta_y \frac{\kappa_{\tilde{y}}}{\tilde{y}+\kappa_{\tilde{y}}} - (\mu+\lambda_y)\, y. \tag{25}$$

The optimizer dynamics are modified by swapping u_1 and u_2 in (17) to implement minimization of y, which is equivalent to the maximization of $\tilde{y}$ since at an equilibrium we would have

$$\tilde{y} = \frac{F(x)}{\mu+\lambda_y}, \qquad y = \frac{\beta_y}{\mu+\lambda_y}\frac{\kappa_{\tilde{y}}}{\tilde{y}+\kappa_{\tilde{y}}}.$$

Fig. 7d When y is activated by $x_1, x_2, \ldots, x_N$, its dynamics are given by (18) where $F(x)$ characterizes the application-specific form of how $x = (x_1,\ x_2,\ \ldots,\ x_N)$ affects the expression of y. Here, we consider the multidimensional generalization of (2) given by

$$F(x) = F_0 e^{-\frac{1}{2}(x-x^*)^\top \Sigma^{-1}(x-x^*)}, \tag{26}$$

$$x^* = \begin{pmatrix} x_1^* \\ x_2^* \\ \vdots \\ x_N^* \end{pmatrix}, \qquad \Sigma = \begin{bmatrix} \sigma_1^2 & \rho_{1,2}\sigma_1\sigma_2 & \cdots & \rho_{1,N}\sigma_1\sigma_N \\ \rho_{2,1}\sigma_2\sigma_1 & \sigma_2^2 & \cdots & \rho_{2,N}\sigma_2\sigma_N \\ \vdots & \vdots & \ddots & \vdots \\ \rho_{N,1}\sigma_N\sigma_1 & \rho_{N,2}\sigma_N\sigma_2 & \cdots & \sigma_N^2 \end{bmatrix}, \tag{27}$$

where $\rho_{i,j} = \rho_{j,i}$ for $i, j \in \{1, 2, \ldots, N\}$ such that $i \neq j$.

The multidimensional optimizer relies on two oscillators: a faster one for generating the "discrete steps" along each dimension (phase selector), and a slower one for activating the dimensions one-by-one (dimension selector). Dynamics of the former are detailed in (10), whereas that of the latter with repressors p_i co-expressed with activators s_i are given by

$$\begin{aligned} \dot{p}_i &= \beta_p \frac{K_p^{n_p}}{p_j^{n_p} + K_p^{n_p}} - (\mu + \lambda_p)\, p_i, \\ \dot{s}_i &= \beta_s \frac{K_p^{n_p}}{p_j^{n_p} + K_p^{n_p}} - (\mu + \lambda_s)\, s_i, \end{aligned} \tag{28}$$

for $i = 1, 2, \ldots, N$ where $j = i - 1$ for $i > 1$ and $j = N$ for $i = 1$. For the data in Fig. 7d, parameters of the dimension selector are chosen so that the period is approximately 10 h ($\beta_p = \beta_s = 20$ μM h^{-1}, $K_p = 1$ nM, $\lambda_p = \lambda_s = 1.1$ h^{-1}, and $n_p = n_s = 4$), corresponding to the experimentally observed 14 generations (*90*).

Modules related to y are shared across all dimensions. Therefore, delay of y to obtain y_d and their comparison yielding y_-, y_+, $\bar{y}_-$ and $\bar{y}_+$ occur according to (9), (12), and (14). Complementing these common signals shared by each scalar optimizer, for each dimension we need separate components for the corresponding regulator x_i: delay modules with dynamics

$$\dot{x}_d^{(i)} = \beta_{x_d} \frac{x_i}{x_i + K_x} - (\mu + \lambda_d)\, x_d^{(i)},$$

comparison modules evolving according to

$$\begin{aligned} \dot{x}_-^{(i)} =& \alpha_x \frac{K_c^{n_r}}{r^{n_r} + K_c^{n_r}} \frac{x_d^{(i)}}{x_d^{(i)} + \bar{K}_x} + \bar{\alpha}_x \frac{a^{n_a}}{a^{n_a} + K_c^{n_a}} \frac{1}{1 + (x_+^{(i)}/\bar{k}_x)^2} - (\mu + \delta_c)\, x_-^{(i)}, \\ \dot{x}_+^{(i)} =& \alpha_x \frac{K_c^{n_r}}{r^{n_r} + K_c^{n_r}} \frac{x_i}{x_i + \bar{K}_x} + \bar{\alpha}_x \frac{a^{n_a}}{a^{n_a} + K_c^{n_a}} \frac{1}{1 + (x_-^{(i)} + \bar{k}_x)^2} - (\mu + \delta_c)\, x_+^{(i)}, \\ \dot{\bar{x}}_-^{(i)} =& \bar{\alpha}_x \frac{a^{n_a}}{a^{n_a} + K_c^{n_a}} \frac{1}{1 + (x_+^{(i)}/\bar{k}_x)^2} - (\mu + \delta_c)\, \bar{x}_-^{(i)}, \\ \dot{\bar{x}}_+^{(i)} =& \bar{\alpha}_x \frac{a^{n_a}}{a^{n_a} + K_c^{n_a}} \frac{1}{1 + (x_-^{(i)}/\bar{k}_x)^2} - (\mu + \delta_c)\, \bar{x}_+^{(i)}, \end{aligned}$$

and logic modules given by

$$\begin{aligned}
\dot{q}_{++}^{(i)} =&\alpha_q \frac{\bar{x}_{+}^{(i)}}{\bar{x}_{+}^{(i)}+k_x'} \frac{\bar{y}_{+}}{\bar{y}_{+}+k_y'} - (\mu+\delta_q)\, q_{++}^{(i)},\\
\dot{q}_{--}^{(i)} =&\alpha_q \frac{\bar{x}_{-}^{(i)}}{\bar{x}_{-}^{(i)}+k_x'} \frac{\bar{y}_{-}}{\bar{y}_{-}+k_y'} - (\mu+\delta_q)\, q_{--}^{(i)},\\
\dot{q}_{+-}^{(i)} =&\alpha_q \frac{\bar{x}_{+}^{(i)}}{\bar{x}_{+}^{(i)}+k_x'} \frac{\bar{y}_{-}}{\bar{y}_{-}+k_y'} - (\mu+\delta_q)\, q_{+-}^{(i)},\\
\dot{q}_{-+}^{(i)} =&\alpha_q \frac{\bar{x}_{-}^{(i)}}{\bar{x}_{-}^{(i)}+k_x'} \frac{\bar{y}_{+}}{\bar{y}_{+}+k_y'} - (\mu+\delta_q)\, q_{-+}^{(i)},\\
\dot{u}_{1}^{(i)} =&\beta_u \bar{s}_i \left(\frac{q_{++}^{(i)}}{q_{++}^{(i)}+k_q} + \frac{q_{--}^{(i)}}{q_{--}^{(i)}+k_q} \right) - (\mu+\lambda_u)\, u_1^{(i)},\\
\dot{u}_{2}^{(i)} =&\beta_u \bar{s}_i \left(\frac{q_{+-}^{(i)}}{q_{+-}^{(i)}+k_q} + \frac{q_{-+}^{(i)}}{q_{-+}^{(i)}+k_q} \right) - (\mu+\lambda_u)\, u_2^{(i)},
\end{aligned}$$

alongside with the regulator and protease dynamics

$$\begin{aligned}
\dot{x}_i =&\beta_x \frac{x_i}{x_i+K_x} + \bar{\beta}_x \frac{(u_1^{(i)}/K_{u_1})^2}{1+(u_1^{(i)}/K_{u_1})^2} - \left[\mu + \lambda_x\left(1+\nu v_i\right)\right] x_i,\\
\dot{v}_i =&\beta_v \frac{u_2^{(i)}}{u_2^{(i)}+K_{u_2}} - (\mu+\lambda_v)\, v_i,
\end{aligned}$$

where the only modification is the inclusion of the term $\bar{s}_i = (s_i/K_s')^{n_s}/[1+(s_i/K_s')^{n_s}]$ in the production of $u_1^{(i)}$ and $u_2^{(i)}$ to ensure that regulation of x_i only occurs when s_i is being expressed during the corresponding cycle of the dimension selector oscillator, otherwise x_i remains approximately constant since $\beta_x \approx K_x(\mu+\lambda_x)$.

Fig. 8 Let s, p, and y denote the concentration of SpoTH, (p)ppGpp, and the reporter protein being expressed from an rrn P1 promoter to serve as a proxy for translational resources (thus for growth rate), respectively. Growth rate regulation via (p)ppGpp occurs according to the simplified dynamics

$$\begin{aligned}
\dot{p} =&\beta_p - (\mu+\lambda_p+\omega s)\, p,\\
\dot{y} =&\beta_y \frac{1}{1+p/\eta_p} \frac{1}{1+s/\eta_s} - (\mu+\lambda_y)\, y,
\end{aligned} \tag{29}$$

together with SpoTH expression

$$\dot{s} = \beta_s \frac{x}{x + \kappa_x} - (\mu + \lambda_s)\, s, \tag{30}$$

where β_s, β_p, and β_y are production rate constants; λ_s, λ_p, and λ_y are degradation rate constants; ω characterizes how fast SpoTH converts (p)ppGpp into GTP/GDP; η_p and η_s capture the negative impact of (p)ppGpp and SpoTH overexpression on the activity of rrn P1 promoters, respectively. This modeling framework is not meant to be comprehensive, instead it aims to capture the main driving forces underpinning growth rate regulation via (p)ppGpp, similarly to the approach taken in (*114*).

To capture the impact of stochastic noise, we replace the ODEs in (29)–(30) with

$$\begin{aligned}
\dot{p} =& \beta_p - (\mu + \lambda_p + \omega s)\, p + \rho\beta_p\xi_p, \\
\dot{y} =& \beta_y \frac{1}{1 + p/\eta_p} \frac{1}{1 + 1/\eta_s} - (\mu + \lambda_y)\, y + \rho\beta_y\xi_y, \\
\dot{s} =& \beta_s \frac{x}{x + \kappa_x} - (\mu + \lambda_s)\, s + \rho\beta_s\xi_s,
\end{aligned} \tag{31}$$

where ρ regulates the intensity of the zero-mean δ-correlated Gaussian white noise ξ_w for $w \in \{p, y, s\}$. Similarly, we replace (9)–(17) governing the dynamics of the optimizer with

$$\begin{aligned}
\dot{x} =& \beta_x \frac{x}{x + K_x} + \bar{\beta}_x \frac{u_1^2}{u_1^2 + K_{u_1}^2} - [\mu + \lambda_x (1 + \nu v)]\, x + \rho\beta_x\xi_x, \\
\dot{v} =& \beta_v \frac{u_2}{u_2 + K_{u_2}} - (\mu + \lambda_v)\, v + \rho\beta_v\xi_v, \\
\dot{x}_d =& \beta_{x_d} \frac{x}{x + K_x} - (\mu + \lambda_d)\, x_d + \rho\beta_{x_d}\xi_{x_d}, \\
\dot{y}_d =& \beta_{y_d} \frac{y}{y + K_y} - (\mu + \lambda_d)\, y_d + \rho\beta_{y_d}\xi_{y_d}, \\
\dot{o}_1 =& \beta_o \frac{1}{1 + (o_3/K_o)^{n_{o_3}}} - (\mu + \lambda_o)\, o_1 + \rho\beta_o\xi_{o_1}, \\
\dot{o}_2 =& \beta_o \frac{1}{1 + (o_1/K_o)^{n_{o_1}}} - (\mu + \lambda_o)\, o_2 + \rho\beta_o\xi_{o_2}, \\
\dot{o}_3 =& \beta_o \frac{1}{1 + (o_2/K_o)^{n_{o_2}}} - (\mu + \lambda_o)\, o_3 + \rho\beta_o\xi_{o_3}, \\
\dot{a} =& \beta_o' \frac{1}{1 + (o_2/K_o)^{n_{o_2}}} - (\mu + \lambda_o)\, a + \rho\beta_o'\xi_a,
\end{aligned} \tag{32}$$

$$
\begin{aligned}
\dot{x}_- =&\alpha_x \frac{K_c^{n_r}}{r^{n_r}+K_c^{n_r}} \frac{x_d}{x_d+\bar{K}_x} + \bar{\alpha}_x \frac{a^{n_a}}{a^{n_a}+K_c^{n_a}} \frac{\bar{k}_x^2}{x_+^2+\bar{k}_x^2} - (\mu+\delta_c)\, x_- + \rho\bar{\alpha}_x \xi_{x_-},\\
\dot{x}_+ =&\alpha_x \frac{K_c^{n_r}}{r^{n_r}+K_c^{n_r}} \frac{x}{x+\bar{K}_x} + \bar{\alpha}_x \frac{a^{n_a}}{a^{n_a}+K_c^{n_a}} \frac{\bar{k}_x^2}{x_-^2+\bar{k}_x^2} - (\mu+\delta_c)\, x_+ + \rho\bar{\alpha}_x \xi_{x_+},\\
\dot{y}_- =&\alpha_y \frac{K_c^{n_r}}{r^{n_r}+K_c^{n_r}} \frac{y_d}{y_d+\bar{K}_y} + \bar{\alpha}_y \frac{a^{n_a}}{a^{n_a}+K_c^{n_a}} \frac{\bar{k}_y^2}{y_+^2+\bar{k}_y^2} - (\mu+\delta_c)\, y_- + \rho\bar{\alpha}_y \xi_{y_-},\\
\dot{y}_+ =&\alpha_y \frac{K_c^{n_r}}{r^{n_r}+K_c^{n_r}} \frac{y}{y+\bar{K}_y} + \bar{\alpha}_y \frac{a^{n_a}}{a^{n_a}+K_c^{n_a}} \frac{\bar{k}_y^2}{y_-^2+\bar{k}_y^2} - (\mu+\delta_c)\, y_+ + \rho\bar{\alpha}_y \xi_{y_+},\\
\dot{\bar{x}}_- =&\bar{\alpha}_x \frac{a^{n_a}}{a^{n_a}+K_c^{n_a}} \frac{\bar{k}_x^2}{x_+^2+\bar{k}_x^2} - (\mu+\delta_c)\, \bar{x}_- + \rho\bar{\alpha}_x \xi_{\bar{x}_-},\\
\dot{\bar{x}}_+ =&\bar{\alpha}_x \frac{a^{n_a}}{a^{n_a}+K_c^{n_a}} \frac{\bar{k}_x^2}{x_-^2+\bar{k}_x^2} - (\mu+\delta_c)\, \bar{x}_+ + \rho\bar{\alpha}_x \xi_{\bar{x}_+},\\
\dot{\bar{y}}_- =&\bar{\alpha}_y \frac{a^{n_a}}{a^{n_a}+K_c^{n_a}} \frac{\bar{k}_y^2}{\bar{y}_+^2+\bar{k}_y^2} - (\mu+\delta_c)\, \bar{y}_- + \rho\bar{\alpha}_y \xi_{\bar{y}_-},\\
\dot{\bar{y}}_+ =&\bar{\alpha}_y \frac{a^{n_a}}{a^{n_a}+K_c^{n_a}} \frac{\bar{k}_y^2}{\bar{y}_-^2+\bar{k}_y^2} - (\mu+\delta_c)\, \bar{y}_+ + \rho\bar{\alpha}_y \xi_{\bar{y}_+},\\
\dot{q}_{++} =&\alpha_q \frac{\bar{x}_+}{\bar{x}_+ + k'_x} \frac{\bar{y}_+}{\bar{y}_+ + k'_y} - (\mu+\delta_q)\, q_{++} + \rho\alpha_q \xi_{q_{++}},\\
\dot{q}_{--} =&\alpha_q \frac{\bar{x}_-}{\bar{x}_- + k'_x} \frac{\bar{y}_-}{\bar{y}_- + k'_y} - (\mu+\delta_q)\, q_{--} + \rho\alpha_q \xi_{q_{--}},\\
\dot{q}_{+-} =&\alpha_q \frac{\bar{x}_+}{\bar{x}_+ + k'_x} \frac{\bar{y}_-}{\bar{y}_- + k'_y} - (\mu+\delta_q)\, q_{+-} + \rho\alpha_q \xi_{q_{+-}},\\
\dot{q}_{-+} =&\alpha_q \frac{\bar{x}_-}{\bar{x}_- + k'_x} \frac{\bar{y}_+}{\bar{y}_+ + k'_y} - (\mu+\delta_q)\, q_{-+} + \rho\alpha_q \xi_{q_{-+}},\\
\dot{u}_1 =&\beta_u \left(\frac{q_{++}}{q_{++}+k_q} + \frac{q_{--}}{q_{--}+k_q} \right) - (\mu+\lambda_u)\, u_1 + \rho\alpha_u \xi_{u_1},\\
\dot{u}_2 =&\beta_u \left(\frac{q_{+-}}{q_{+-}+k_q} + \frac{q_{-+}}{q_{-+}+k_q} \right) - (\mu+\lambda_u)\, u_2 + \rho\alpha_u \xi_{u_2}.
\end{aligned} \tag{33}
$$

Data in Supplementary Fig. 16 highlight that closed loop performance is robust to stochastic noise, even when noise intensity is sufficient to result in completely absent periods of the phase selector, and in random switches between the two stable equilibria of the toggle switches. Furthermore, the optimizer successfully tracks the time-varying optimum both when the shifts in its location are gradual and when they are abrupt, illustrated in Supplementary Fig. 17.

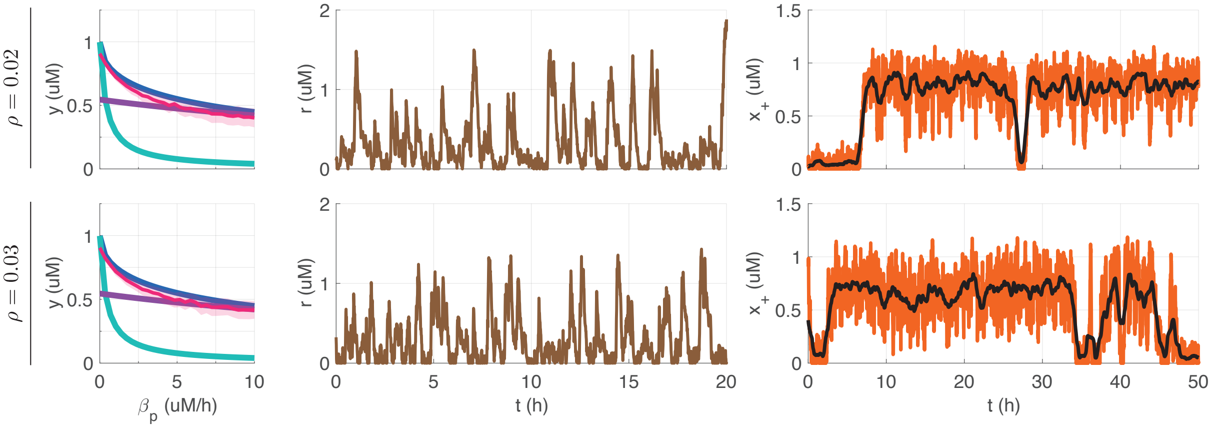

Supplementary Fig. 16. **Closed loop performance of (p)ppGpp mediated growth regulation is robust to stochastic noise.** Parameters of the optimizer module are taken from Supplementary Table 1, together with $\beta_s = 1\,\mu\text{M h}^{-1}$, $\beta_y = 2\,\mu\text{M h}^{-1}$, $\lambda_s = 5\,\text{h}^{-1}$, $\lambda_p = 20\,\text{h}^{-1}$, $\kappa_x = 500$ nM, $\eta_p = 20$ nM, $\eta_s = 200$ nM, and $\omega = 20\,\text{nM}^{-1}\,\text{h}^{-1}$. Stochastic simulations are carried out using an Euler-Maruyama scheme (*115*) with a step size of $\Delta t = 0.01$ h for $T = 100$ h considering 100 independent simulations with random initial conditions. Performance is evaluated during the second half of each simulation by considering the average of y and its standard deviation. Red curves and shaded regions denote the mean and standard deviation of these averages, respectively. Blue lines denote the optimum that can be achieved by maximizing y via s at the steady state of (29). Green and purple correspond to the case with zero and maximal induction of s. Black curves denote 1 h moving average. Trajectories depict the behavior of isolated modules (repressilator and toggle switch). In the absence of noise, the repressilator displays regular oscillations (Supplementary Fig. 7) and the toggle switch remains at the equilibrium based on the initial condition. In the presence of stochastic noise, oscillations become irregular and random transitions appear between stable fixed points of the switch.

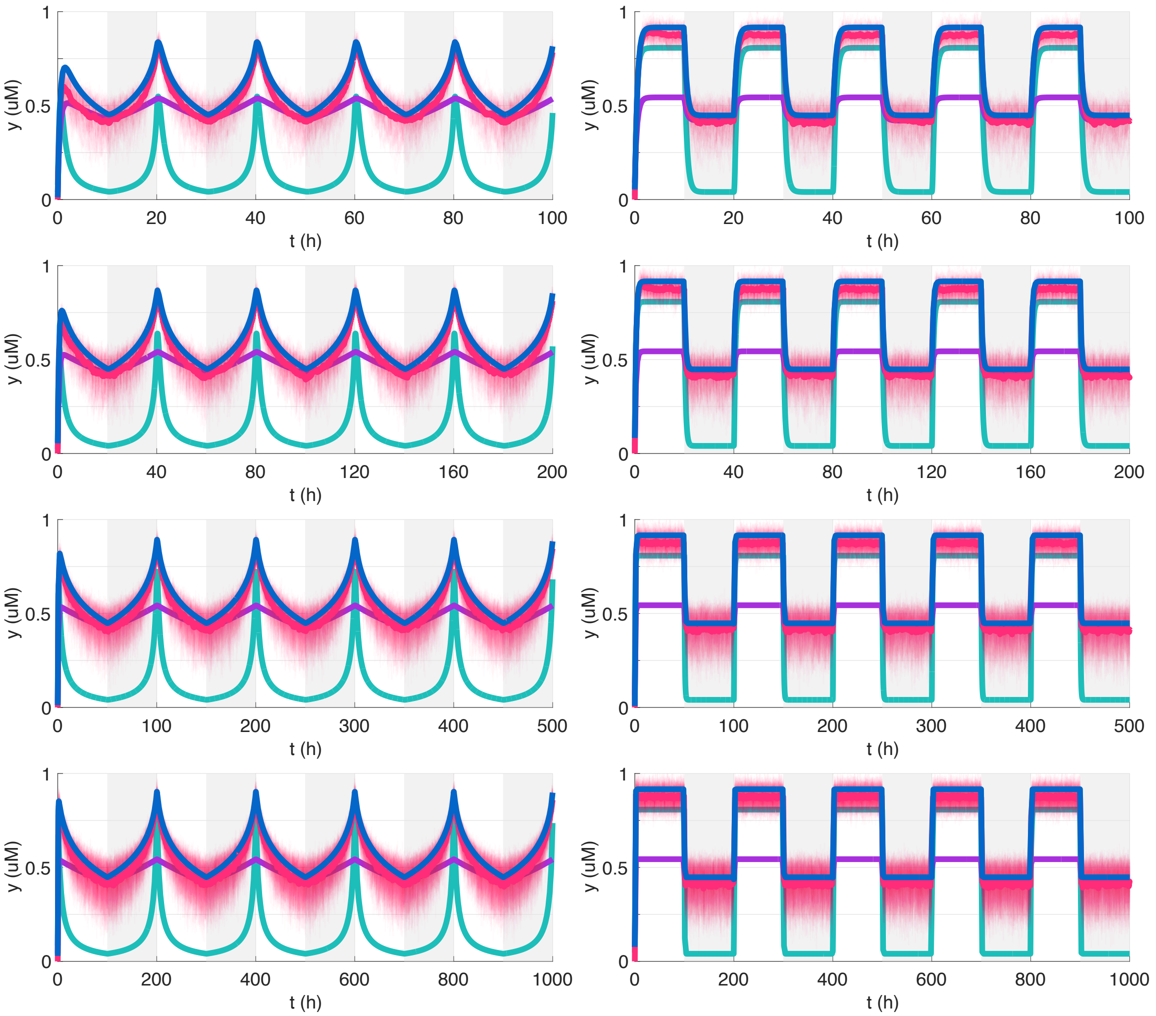

Supplementary Fig. 17. **The optimizer successfully tracks the time-varying optimum.** Parameters of the optimizer module are taken from Supplementary Table 1, together with $\beta_s = 1\,\mu\text{M h}^{-1}$, $\beta_y = 2\,\mu\text{M h}^{-1}$, $\lambda_s = 5\,\text{h}^{-1}$, $\lambda_p = 20\,\text{h}^{-1}$, $\kappa_x = 500$ nM, $\eta_p = 20$ nM, $\eta_s = 200$ nM, and $\omega = 20\,\text{nM}^{-1}\,\text{h}^{-1}$. Stochastic simulations of the closed loop system with the optimizer are carried out using an Euler-Maruyama scheme (*115*) with a step size of $\Delta t = 0.01$ h and noise intensity $\rho = 0.03$ considering 100 independent simulations with random initial conditions, denoted by light red curves, dark red curves correspond to their temporal averages. Blue lines denote the optimum that can be achieved by maximizing y via s at the steady state of (29). Green and purple correspond to the case with zero and maximal induction of s. Cellular stress is modulated periodically via β_p as in Fig. 8d, the period increases 10-fold from the top row to the bottom row without considerable change in closed loop performance.

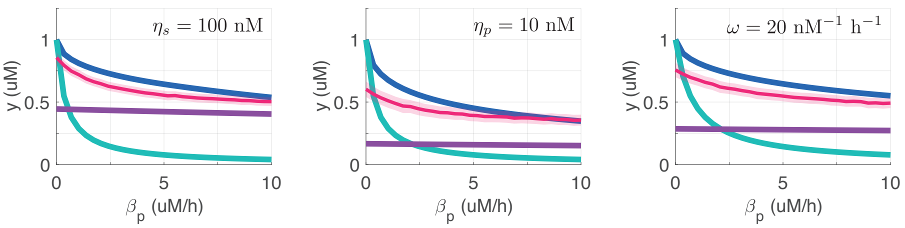

Supplementary Fig. 18. **Closed loop performance of (p)ppGpp mediated growth regulation is robust to parameter variations.** Parameters of the optimizer module are taken from Supplementary Table 1, together with $\beta_s = 1\ \mu\text{M h}^{-1}$, $\beta_y = 2\ \mu\text{M h}^{-1}$, $\lambda_s = 5\ \text{h}^{-1}$, $\lambda_p = 20\ \text{h}^{-1}$, $\kappa_x = 500$ nM, $\eta_p = 20$ nM, $\eta_s = 200$ nM, and $\omega = 20\ \text{nM}^{-1}\ \text{h}^{-1}$, unless otherwise indicated in the figure. Stochastic simulations of the closed loop system with the optimizer are carried out using an Euler-Maruyama scheme (*115*) with a step size of $\Delta t = 0.01$ h and noise intensity $\rho = 0.03$ for $T = 100$ h considering 100 independent simulations with random initial conditions. Performance is evaluated during the second half of each simulation by considering the average of y and its standard deviation. Red curves and shaded regions denote the mean and standard deviation of these averages, respectively. Blue lines denote the optimum that can be achieved by maximizing y via s at the steady state of (29). Green and purple correspond to the case with zero and maximal induction of s.

Selected values of the design parameters β_s, β_y, κ_x, λ_s, λ_y fall within the typical ranges in *E. coli* (Supplementary Section 2.2). Other parameters in (31) are instead part of the natural (p)ppGpp-mediated pathway of growth control. Regarding λ_p, (p)ppGpp is reported to have a half-life of approximately 1–3 minutes (*116–118*). Since the simplified dynamics in (29)–(30) of this pathway are not a comprehensive mechanistic representation, exact values of ω, η_p, and η_s are difficult to estimate. Importantly, closed loop performance is robust to changes in these parameters (Supplementary Fig. 18) without requiring additional tuning of the optimizer itself.

4 Metabolic burden

To estimate the bioenergetic cost of the optimizer, we consider two reference circuits: the 3-input and 4-input AND gates implemented in (*119*), the former with 3, 6, and 2 genes harbored in plasmids equipped with pSC101, p15A, and pColE1 origins of replication, respec-

tively, whereas the latter with 4 additional genes in the first plasmid. As the plasmid copy numbers are 4 for pSC101, 11 for p15A, and 15 for pColE1 (*97*), total load of the reference circuits is approximately equivalent to that of $3 \times 4 + 6 \times 11 + 2 \times 15 = 108$ genes and $7 \times 4 + 6 \times 11 + 2 \times 15 = 124$ genes, expressed from a single copy of DNA (e.g., host chromosome). With an average protein production rate constant $\beta = 1$ μM h^{-1} (Supplementary Section 2.2), the total protein production rate constants are thus approximately 108 μM h^{-1} and 124 μM h^{-1}, respectively.

Considering the parameter values featured in Supplementary Table 1, the protein-based components of the optimizer have an equivalent protein production rate constant of 143 μM h^{-1}, approximately 30% and 15% greater than those of the reference circuits. Given that over 50% of this cost is due to the repressilator, this burden can be significantly decreased by reducing the production rate constant β_o. For instance, changing it from 20 μM h^{-1} to 5 μM h^{-1} (e.g., by integrating the repressilator into the chromosome instead of harboring it on a plasmid with pSC101 origin of replication) reduces the total protein production rate constant over 40%, from approximately 143 μM h^{-1} to 83 μM h^{-1}, without affecting closed loop performance (Supplementary Fig. 19). Even when considering an implementation with two separate and compatible plasmids with low copy number (e.g., pSC101 and TULIP (*53*)) and the corresponding antibiotic resistance genes, the total protein production rate constant remains approximately 90 μM h^{-1}, about 10% and 30% lower than those of the reference circuits. Performance is identical if δ_o is also adjusted to maintain the 2 h cycle length of the phase selector oscillator (Supplementary Fig. 20).

As growth is primarily limited by the availability of translational resources (*120, 121*), even with the RNA-based components we expect the metabolic burden of the optimizer to be comparable to that of the reference circuits. Since the reference circuits had no negative impact on doubling time (*119*), we thus expect that the proposed optimizer can be deployed without

significantly affecting growth rate (as long as the toxicity threshold of individual proteins are not exceeded (*122*)). In all the calculations above, the total production rate constants are upper estimates as not all species are constantly expressed. However, this approximation error is likely similar across circuits, thus we expect the comparison to be reasonable.

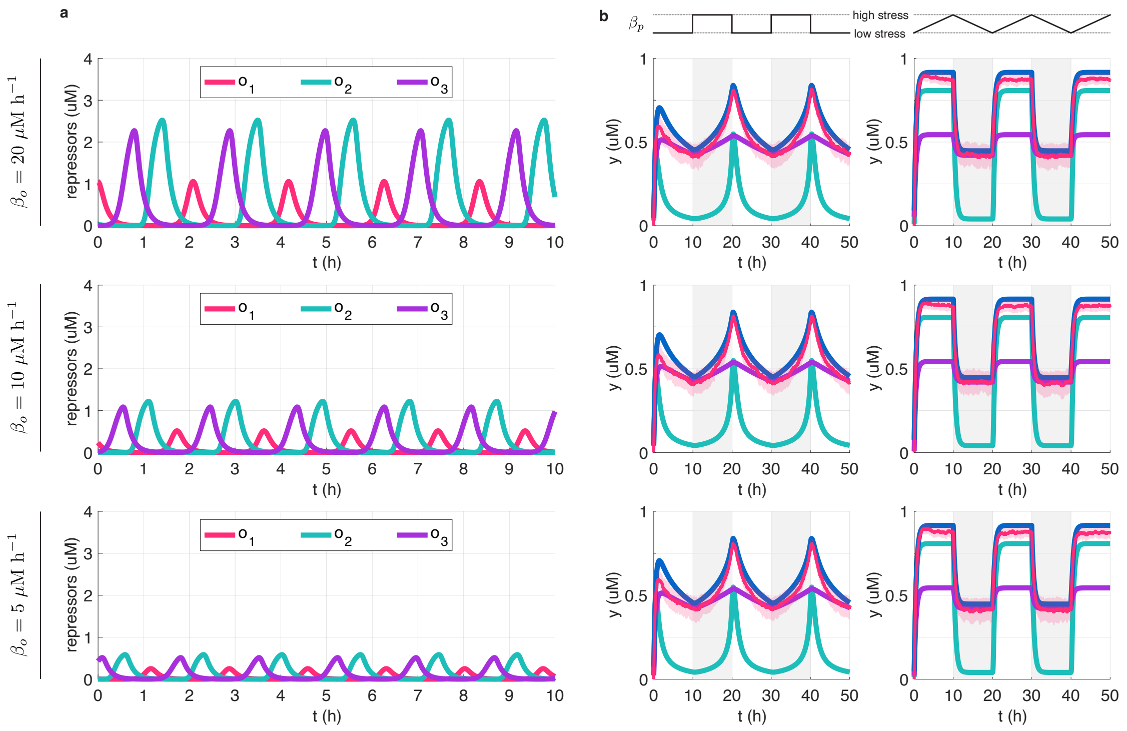

Supplementary Fig. 19. **Metabolic burden of the optimizer can be reduced without negatively impacting closed loop performance (only β_o is reduced).** Parameters of the optimizer module are taken from Supplementary Table 1, except for β_o. Parameters of the (p)ppGpp pathway remain unchanged, that is, $\beta_s = 1\ \mu\text{M h}^{-1}$, $\beta_y = 2\ \mu\text{M h}^{-1}$, $\lambda_s = 5\text{ h}^{-1}$, $\lambda_p = 20\text{ h}^{-1}$, $\kappa_x = 500$ nM, $\eta_p = 20$ nM, $\eta_s = 200$ nM, and $\omega = 20\text{ nM}^{-1}\text{ h}^{-1}$. **a** Behavior of the repressilator (in the absence of stochastic noise). **b** Closed loop performance in the presence of time-varying stress modulated via β_p, as discussed in case of Fig. 8d. Stochastic simulations of the closed loop system with the optimizer are carried out using an Euler-Maruyama scheme (*115*) with a step size of $\Delta t = 0.01$ h and noise intensity $\rho = 0.03$ considering 100 independent simulations with random initial conditions. Red curves and shaded regions denote the mean and standard deviation of these trajectories, respectively. Blue lines denote the optimum that can be achieved by maximizing y via s at the steady state of (29). Green and purple correspond to the case with zero and maximal induction of s.

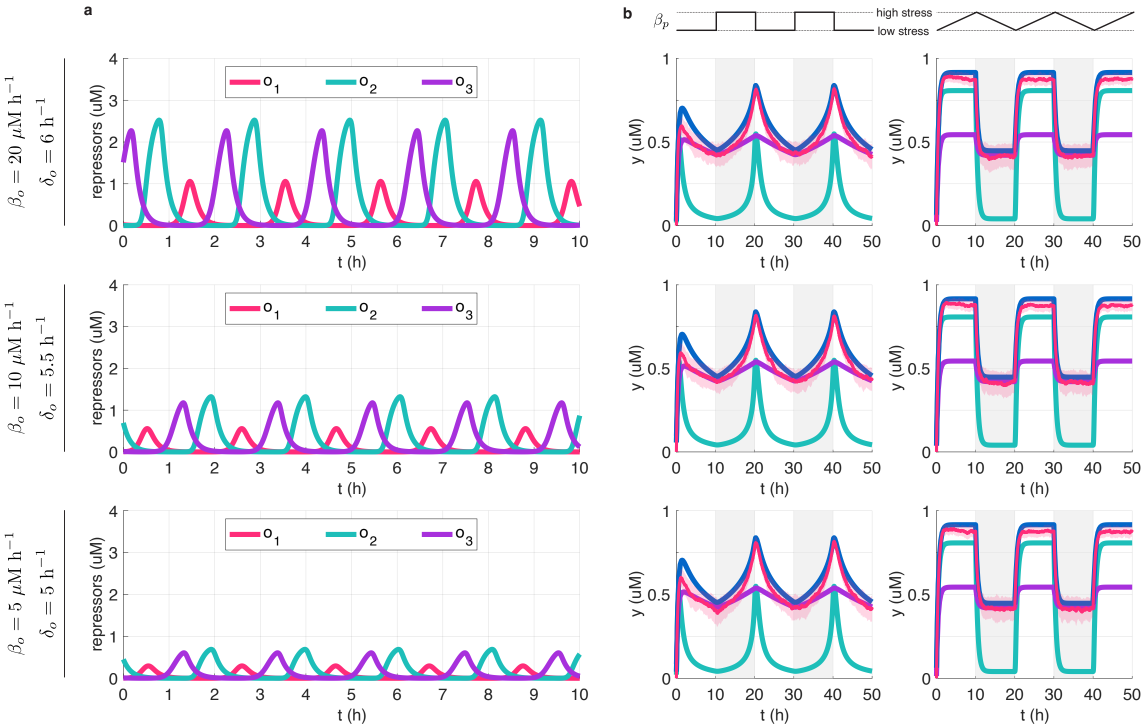

Supplementary Fig. 20. **Metabolic burden of the optimizer can be reduced without negatively impacting closed loop performance (both β_o and δ_o are reduced).** Parameters of the optimizer module are taken from Supplementary Table 1, except for β_o and δ_o. Parameters of the (p)ppGpp pathway remain unchanged, that is, $\beta_s = 1\ \mu\text{M h}^{-1}$, $\beta_y = 2\ \mu\text{M h}^{-1}$, $\lambda_s = 5\ \text{h}^{-1}$, $\lambda_p = 20\ \text{h}^{-1}$, $\kappa_x = 500$ nM, $\eta_p = 20$ nM, $\eta_s = 200$ nM, and $\omega = 20\ \text{nM}^{-1}\ \text{h}^{-1}$. **a** Behavior of the repressilator (in the absence of stochastic noise). **b** Closed loop performance in the presence of time-varying stress modulated via β_p, as discussed in case of Fig. 8d. Stochastic simulations of the closed loop system with the optimizer are carried out using an Euler-Maruyama scheme (*115*) with a step size of $\Delta t = 0.01$ h and noise intensity $\rho = 0.03$ considering 100 independent simulations with random initial conditions. Red curves and shaded regions denote the mean and standard deviation of these trajectories, respectively. Blue lines denote the optimum that can be achieved by maximizing y via s at the steady state of (29). Green and purple correspond to the case with zero and maximal induction of s.

5 Simulation details

Fig. 1 The objective function takes the form of (2). In panel (b), $\sigma = x^*$ and the time-varying optimum x^* is depicted in blue, together with $\epsilon_y = 0$. The initial conditions $x(0)$ are equidistantly spaced over the interval $[0, 1]$ with 20 sample points. In panel (c), $\epsilon_x = 100$, $\sigma = x^*/10$, the time varying optimum x^* is depicted in blue. In the top row $t_d = 1$, in the bottom row $t_d = 10$. In the left column $\epsilon_y = 0.1$, in the right column $\epsilon_y = 1$.

Fig. 2 The objective function takes the form of (2) with $\sigma = x^*/3$, the time varying optimum x^* is depicted in blue. Across all panels $\epsilon_x = 100$. The panel in the top left corner corresponds to $\epsilon_y = \epsilon_x/100$, and ϵ_y increases towards the lower right panel where $\epsilon_y = \epsilon_x/10$ (sample points are spaced equidistantly on a logarithmic scale), and $\epsilon_d = \epsilon_y/2$ for light red, $\epsilon_d = \epsilon_y$ for medium red, and $\epsilon_d = 2\epsilon_y$ for dark red.

Fig. 3 The objective function takes the form of (2) with $\sigma = x^*/3$, the time varying optimum x^* is depicted in blue, together with $\epsilon_x = 100$, $\epsilon_y = 1$, $\epsilon_d = 10$, $\epsilon_c = 0.1$, $n = 2$, $\alpha_{c,1} = 1$, and $\alpha_{c,2} = 10$ unless otherwise indicated. A square wave signal is used with period $\tau = 10$ and duty cycle 10% for the oscillator in the comparator.

Fig. 4 The objective function takes the form of (2) with $\sigma = x^*/3$, the time varying optimum x^* is depicted in blue, together with $\epsilon_x = 100$, $\epsilon_y = 1$, $\epsilon_d = 10$, $\epsilon_c = \epsilon_l = 0.1$, $n = 2$ and $\alpha_{c,1} = 1$ in all plots. A square wave signal is used with period $\tau = 10$ and duty cycle 10% for the oscillator in the comparator. In case of red, green, purple, and orange, the value of $\alpha_{c,2}$ in the comparator module is 10, 30, 50, 150, respectively, alongside with the dissociation constants $(K_\wedge, K_\vee) = (1.5817, 0.1062)$, $(K_\wedge, K_\vee) = (0.5066, 0.0381)$ $(K_\wedge, K_\vee) = (2.1865, 0.0233)$, and $(K_\wedge, K_\vee) = (0.3671, 0.0271)$.

Fig. 5 In all plots, the objective function takes the form of (2) and the time varying optimum x^* is depicted in blue. In panel (a), $\sigma = x^*/3$, the duty cycle of the oscillator in the comparator is 10%, together with $\epsilon_y = 0$, $\epsilon_c = 0.01$, $\alpha_{c,1} = 1$, $\alpha_{c,2} = 100$, $n = 2$, $K_\wedge = \sqrt{\alpha_c}$, $K_\vee = 1/K_\wedge$, $\epsilon_d = 0.5$ for light/dark red, and $\epsilon_d = 5$ for light/dark green. From left to right, the period τ of the oscillator in the comparator module is 10, 10, 10, and 5, whereas the the value of ϵ_x is 100, 150, 200, 200, respectively. Stochastic simulations are carried out considering $\epsilon_x \dot{x} = u_1 - u_2 x + \sigma\xi$ where σ regulates the intensity of the zero-mean δ-correlated Gaussian white noise ξ, using an Euler-Maruyama scheme (*115*) with 200,000 equal time steps and noise intensity $\sigma = 1$. In panel (b), $\sigma = x^*/10$, the duty cycle of the oscillator in the comparator is 10% and its period is $\tau = 5$, together with $\epsilon_x = 100$, $\epsilon_y = 0$, $\epsilon_d = 1$, and $\epsilon_c = 0.01$, $\alpha_{c,1} = 1$, $\alpha_{c,2} = 100$, $n = 2$, $K_\wedge = \sqrt{\alpha_c}$, and $K_\vee = 1/K_\wedge$. The value of α_d for tracking y with y_d is 0.9, 0.95, 1.05, and 1.1 for light green, dark green, dark red, light red, respectively. In panel (c), $\sigma = x^*/10$, the duty cycle of the oscillator in the comparator is 10% and its period is $\tau = 5$, together with $\epsilon_x = 100$, $\epsilon_y = 0$, $\epsilon_d = 1$, and $\epsilon_c = 0.01$, $\alpha_d = 1$, $\alpha_{c,2} = 100$, $n = 2$, $K_\wedge = \sqrt{\alpha_c}$, and $K_\vee = 1/K_\wedge$. The value of $\alpha_{c,1}$ for y_- and y_+ is $(1.05, 0.95)$ and $(1.005, 0.995)$ for red and green, respectively.

Fig. 7 In panels (a)–(c), parameters of the optimizer module are taken from Supplementary Table 1. In panel (d), all scalar optimizer modules are identical with parameters taken from Supplementary Table 1. In panels (a) and (c), the objective function takes the form (20) with $n_x = 2$, $K_F = 100$ nM, and $F_0 = 100\,\text{nM}\,\text{h}^{-1}$; data were collected for $T = 200$ h, mean and error bars denote the average of x and its standard deviation during the second half of the simulation after the initial transient has disappeared, averaged over 100 independent simulations with randomly selected initial conditions. In panels (b) and (d), results of 30 independent simulations are presented with randomly selected initial conditions. In panel (a), parameters of direct activation are featured in Supplementary Table 1; parameters of the regulatory cascade

without self-activation are $N = 3$, $\beta_z = 5\ \mu\text{M h}^{-1}$, $\kappa_z = 1\ \mu\text{M}$, and $\lambda_z = 4\ \text{h}^{-1}$; and parameters of the regulatory cascade with self-activation are $\beta_{z,1} = 4\ \mu\text{M h}^{-1}$, $\beta_{z,2} = 1.5\ \mu\text{M h}^{-1}$, $\kappa_x = \kappa_z = 1\ \mu\text{M}$, and $\lambda_z = 4\ \text{h}^{-1}$. In panel (b), parameters of the feedback regulated cascade are $\beta_w = \beta_z = 4\ \mu\text{M h}^{-1}$, $\lambda_w = \lambda_z = 3\ \text{h}^{-1}$, and the optimum occurs at $\theta^* = z^* = 50$ nM. When κ_x is modulated from $\kappa_x = 0.5\ \mu\text{M}$ through $\kappa_x = 0.1\ \mu\text{M}$ to $\kappa_x = 1.5\ \mu\text{M}$, we have $\kappa_w = 0.5\ \mu\text{M}$ and $\kappa_z = 0.1\ \mu\text{M}$ throughout the entire simulation. When κ_w is modulated from $\kappa_w = 0.5\ \mu\text{M}$ through $\kappa_w = 1.5\ \mu\text{M}$ to $\kappa_w = 0.1\ \mu\text{M}$, we have $\kappa_x = 0.5\ \mu\text{M}$ and $\kappa_z = 0.1\ \mu\text{M}$ throughout the entire simulation. When κ_z is modulated from $\kappa_z = 0.1\ \mu\text{M}$ through $\kappa_z = 0.02\ \mu\text{M}$ to $\kappa_z = 0.01\ \mu\text{M}$, we have $\kappa_x = 0.5\ \mu\text{M}$ and $\kappa_w = 0.5\ \mu\text{M}$ throughout the entire simulation. In panel (b), parameters of the feedforward loop are $\lambda_z = 2\ \text{h}^{-1}$, $\kappa_w = 10$ nM, $\kappa_z = 200$ nM, and $\beta_y = 50\ \text{nM h}^{-1}$. When κ_x is modulated from $\kappa_x = 70$ nM through $\kappa_x = 10$ nM to $\kappa_x = 40$ nM, we have $\beta_z = 1.2\ \mu\text{M h}^{-1}$ and $w = 0$ nM throughout the entire simulation. When β_z is modulated from $\beta_z = 1.2\ \mu\text{M h}^{-1}$ through $\beta_z = 3.0\ \mu\text{M h}^{-1}$ to $\beta_z = 0.5\ \mu\text{M h}^{-1}$, we have $\kappa_x = 70$ nM and $w = 0$ nM throughout the entire simulation. When w is modulated from $w = 0$ nM through $w = 10$ nM to $w = 5$nM, we have $\kappa_x = 70$ nM and $\beta_z = 1.2\ \mu\text{M h}^{-1}$ throughout the entire simulation. In panel (c), parameters are $\beta_y = 100\ \text{nM h}^{-1}$, $\kappa_{\tilde{y}} = 0.1$ nM, and $\lambda_{\tilde{y}} = 4\ \text{h}^{-1}$. In panel (d), the objective function takes the form (26)–(27), where $F_0 = 100\ \text{nM h}^{-1}$, $\sigma_1 = \sigma_2 = \sigma_3 = 20$ nM, $\rho_{1,2} = \rho_{2,3} = \rho_{1,3} = 0.5$. Parameters of the dimension selector module with dynamics (28) are $\beta_p = \beta_s = 20\ \mu\text{M h}^{-1}$, $\lambda_p = \lambda_s = 1.1\ \text{h}^{-1}$, $K_p = 1$ nM, $K_s = 10$ nM, and $n_p = n_s = 4$.

Fig. 8 Parameters of the optimizer module are taken from Supplementary Table 1, together with $\beta_s = 1\ \mu\text{M h}^{-1}$, $\beta_y = 2\ \mu\text{M h}^{-1}$, $\lambda_s = 5\ \text{h}^{-1}$, $\lambda_p = 20\ \text{h}^{-1}$, $\kappa_x = 500$ nM, $\eta_p = 20$ nM, $\eta_s = 200$ nM, and $\omega = 20\ \text{nM}^{-1}\ \text{h}^{-1}$. Low stress and high stress correspond to $\beta_p = 0.1\ \mu\text{M h}^{-1}$ and $\beta_p = 10\ \mu\text{M h}^{-1}$, respectively. Blue lines denote the optimum that can be

achieved by maximizing y at the steady state of (29) via numerically selecting the optimal value of s. Green and purple correspond to the case with zero and maximal induction of s. Stochastic simulations of the closed loop system (31)–(33) are carried out using an Euler-Maruyama scheme (*115*) with a step size of $\Delta t = 0.01$ h and noise intensity $\rho = 0.03$ for $T = 100$ h. In panel (c), closed loop performance is evaluated based on 100 independent simulations with random initial conditions during the second half of each simulation by considering the average of y and its standard deviation. Red curves denote the mean of these averages, red shaded regions denote the mean of these standard deviations. In panel (d), red curves and shaded regions denote the mean and standard deviation of the closed loop system considering 100 independent simulations with random initial conditions.

References

1. Gyorgy, A. *et al.* Isocost Lines Describe the Cellular Economy of Genetic Circuits. *Biophys J* **109**, 639–646 (2015).

2. Frei, T. *et al.* Characterization and mitigation of gene expression burden in mammalian cells. *Nature Communications* **11**, 4641 (2020).

3. Ceroni, F., Algar, R., Stan, G.-B. & Ellis, T. Quantifying cellular capacity identifies gene expression designs with reduced burden. *Nature Methods* **12**, 415–418 (2015).

4. Butzin, N. C., Hochendoner, P., Ogle, C. T. & Mather, W. H. Entrainment of a Bacterial Synthetic Gene Oscillator through Proteolytic Queueing. *ACS Synthetic Biology* **6**, 455–462 (2017).

5. Gardner, T. S., Cantor, C. R. & Collins, J. J. Construction of a genetic toggle switch in E. coli. *Nature* **403**, 339–42 (2000).

6. Cuba Samaniego, C. & Franco, E. A robust molecular network motif for period-doubling devices. *ACS Synthetic Biology* **7**, 75–85 (2018). PMID: 29227103.

7. Zhu, R., del Rio-Salgado, J. M., Garcia-Ojalvo, J. & Elowitz, M. Synthetic multistability in mammalian cells. *bioRxiv* (2021).

8. Jaruszewicz-Błońska, J. & Lipniacki, T. Genetic toggle switch controlled by bacterial growth rate. *BMC Systems Biology* **11**, 117 (2017).

9. Jaruszewicz, J. & Lipniacki, T. Toggle switch: noise determines the winning gene. *Physical Biology* **10**, 035007 (2013).

10. Pokhilko, A., Ebenhöh, O., Stark, W. M. & Colloms, S. D. Mathematical model of a serine integrase-controlled toggle switch with a single input. *Journal of the Royal Society Interface* **15**, 20180160 (2018).

11. Siegal-Gaskins, D., Franco, E., Zhou, T. & Murray, R. M. An analytical approach to bistable biological circuit discrimination using real algebraic geometry. *Journal of the Royal Society Interface* **12**, 20150288 (2015).

12. Morelli, M. J., Tănase-Nicola, S., Allen, R. J. & ten Wolde, P. R. Reaction Coordinates for the Flipping of Genetic Switches. *Biophysical Journal* **94**, 3413–3423 (2008).

13. Purcell, O., di Bernardo, M., Grierson, C. S. & Savery, N. J. A multi-functional synthetic gene network: A frequency multiplier, oscillator and switch. *PLOS ONE* **6**, 1–12 (2011).

14. Lee, J. W. *et al.* Creating Single-Copy Genetic Circuits. *Molecular Cell* **63**, 329–336 (2016).

15. Li, T. *et al.* Engineering of a genetic circuit with regulatable multistability. *Integrative Biology* **10**, 474–482 (2018).

16. Lyons, S. M., Xu, W., Medford, J. & Prasad, A. Loads bias genetic and signaling switches in synthetic and natural systems. *PLoS Comput Biol* **10**, e1003533 (2014).

17. Tian, T. & Burrage, K. Stochastic models for regulatory networks of the genetic toggle switch. *Proceedings of the National Academy of Sciences* **103**, 8372–8377 (2006).

18. Strasser, M., Theis, F. J. & Marr, C. Stability and Multiattractor Dynamics of a Toggle Switch Based on a Two-Stage Model of Stochastic Gene Expression. *Biophysical Journal* **102**, 19–29 (2012).

19. Yong, C. & Gyorgy, A. Stability and robustness of unbalanced genetic toggle switches in the presence of scarce resources. *Life* **11** (2021).

20. Greco, F. V., Pandi, A., Erb, T. J., Grierson, C. S. & Gorochowski, T. E. Harnessing the central dogma for stringent multi-level control of gene expression. *Nature Communications* **12**, 1738 (2021).

21. Du, M., Kodner, S. & Bai, L. Enhancement of LacI binding in vivo. *Nucleic Acids Research* **47**, 9609–9618 (2019).

22. Bartoli, V., Meaker, G. A., Bernardo, M. d. & Gorochowski, T. E. Tunable genetic devices through simultaneous control of transcription and translation. *Nature Communications* **11**, 2095 (2020).

23. Szydlo, K., Ignatova, Z. & Gorochowski, T. E. Improving the Robustness of Engineered Bacteria to Nutrient Stress Using Programmed Proteolysis. *ACS Synthetic Biology* **11**, 1049–1059 (2022).

24. Cooper, S. & Helmstetter, C. E. Chromosome replication and the division cycle of Escherichia coli B/r. *Journal of molecular biology* **31**, 519–40 (1968).

25. Bremer, H. & Dennis, P. P. Modulation of Chemical Composition and Other Parameters of the Cell by Growth Rate. *Escherichia Coli and Salmonella: Cellular and Molecular Biology* (1996).

26. Liang, S.-T. *et al.* Activities of constitutive promoters in Escherichia coli 1 1 Edited by D. E. Draper. *Journal of Molecular Biology* **292**, 19–37 (1999).

27. Klumpp, S. & Hwa, T. Growth-rate-dependent partitioning of RNA polymerases in bacteria. *Proceedings of the National Academy of Sciences* **105**, 20245–20250 (2008).

28. Bremer, H. & Dennis, P. P. Modulation of Chemical Composition and Other Parameters of the Cell at Different Exponential Growth Rates. *EcoSal Plus* **3** (2008).

29. Reshes, G., Vanounou, S., Fishov, I. & Feingold, M. Timing the start of division in E. coli: a single-cell study. *Physical Biology* **5**, 046001 (2008).

30. Campos, M. *et al.* A Constant Size Extension Drives Bacterial Cell Size Homeostasis. *Cell* **159**, 1433–1446 (2014).

31. Long, C. P., Gonzalez, J. E., Feist, A. M., Palsson, B. O. & Antoniewicz, M. R. Fast growth phenotype of E. coli K-12 from adaptive laboratory evolution does not require intracellular flux rewiring. *Metabolic Engineering* **44**, 100–107 (2017).

32. Milo, R. What is the total number of protein molecules per cell volume? A call to rethink some published values. *BioEssays* **35**, 1050–1055 (2013).

33. Pai, A. & You, L. Optimal tuning of bacterial sensing potential. *Molecular Systems Biology* **5**, 286–286 (2009).

34. Pedersen, S., Reeh, S. & Friesen, J. D. Functional mRNA half lives in E. coli. *Molecular and General Genetics MGG* **166**, 329–336 (1978).

35. Chow, J. & Dennis, P. P. Coupling between mRNA synthesis and mRNA stability in Escherichia coli. *Molecular Microbiology* **11**, 919–931 (1994).

36. Bernstein, J. A., Khodursky, A. B., Lin, P.-H., Lin-Chao, S. & Cohen, S. N. Global analysis of mRNA decay and abundance in Escherichia coli at single-gene resolution using two-color fluorescent DNA microarrays. *PNAS* **99**, 9697–9702 (2002).

37. Taniguchi, Y. *et al.* Quantifying E. coli Proteome and Transcriptome with Single-Molecule Sensitivity in Single Cells. *Science* **329**, 533–538 (2010).

38. Bouvet, P. & Belasco, J. G. Control of RNase E-mediated RNA degradation by 5'-terminal base pairing in E. coil. *Nature* **360**, 488–491 (1992).

39. Baker, K. E. & Mackie, G. A. Ectopic RNase E sites promote bypass of 5'-end-dependent mRNA decay in Escherichia coli. *Molecular Microbiology* **47**, 75–88 (2003).

40. Deana, A., Celesnik, H. & Belasco, J. G. The bacterial enzyme RppH triggers messenger RNA degradation by 5' pyrophosphate removal. *Nature* **451**, 355–358 (2008).

41. Garrey, S. M. & Mackie, G. A. Roles of the 5'-phosphate sensor domain in rnase e. *Molecular Microbiology* **80**, 1613–1624 (2011).

42. Mackie, G. A. RNase E: at the interface of bacterial RNA processing and decay. *Nature Reviews Microbiology* **11**, 45–57 (2013).

43. Chen, H., Shiroguchi, K., Ge, H. & Xie, X. S. Genome-wide study of mRNA degradation and transcript elongation in Escherichia coli. *Molecular Systems Biology* **11**, 781 (2015).

44. Chen, H., Shiroguchi, K., Ge, H. & Xie, X. S. Genome-wide study of mRNA degradation and transcript elongation in Escherichia coli. *Molecular Systems Biology* **11**, 808 (2015).

45. Bernstein, J. A., Khodursky, A. B., Lin, P.-H., Lin-Chao, S. & Cohen, S. N. Global analysis of mRNA decay and abundance in Escherichia coli at single-gene resolution using two-color fluorescent DNA microarrays. *Proceedings of the National Academy of Sciences* **99**, 9697–9702 (2002).

46. Selinger, D. W., Saxena, R. M., Cheung, K. J., Church, G. M. & Rosenow, C. Global RNA Half-Life Analysis in Escherichia coli Reveals Positional Patterns of Transcript Degradation. *Genome Research* **13**, 216–223 (2003).

47. Kennell, D. & Riezman, H. Transcription and translation initiation frequencies of the Escherichia coli lac operon. *Journal of Molecular Biology* **114**, 1–21 (1977).

48. Liang, S.-T., Ehrenberg, M., Dennis, P. & Bremer, H. Decay of rplN and lacZ mRNA in Escherichia coli11Edited by D. E. Draper. *Journal of Molecular Biology* **288**, 521–538 (1999).

49. Bremer, H., Dennis, P. & Ehrenberg, M. Free RNA polymerase and modeling global transcription in Escherichia coli. *Biochimie* **85**, 597–609 (2003).

50. Wade, J. T. & Struhl, K. The transition from transcriptional initiation to elongation. *Current Opinion in Genetics & Development* **18**, 130–136 (2008).

51. Proshkin, S., Rahmouni, A. R., Mironov, A. & Nudler, E. Cooperation Between Translating Ribosomes and RNA Polymerase in Transcription Elongation. *Science* **328**, 504–508 (2010).

52. Gong, X. *et al.* Comparative analysis of essential genes and nonessential genes in Escherichia coli K12. *Molecular Genetics and Genomics* **279**, 87–94 (2008).

53. Joshi, S. H.-N., Yong, C. & Gyorgy, A. Inducible plasmid copy number control for synthetic biology in commonly used E. coli strains. *Nature Communications* **13**, 6691 (2022).

54. Gong, S., Yu, H. H., Johnson, K. A. & Taylor, D. W. DNA Unwinding Is the Primary Determinant of CRISPR-Cas9 Activity. *Cell Reports* **22**, 359–371 (2018).

55. Josephs, E. A. *et al.* Structure and specificity of the RNA-guided endonuclease Cas9 during DNA interrogation, target binding and cleavage. *Nucleic Acids Research* **43**, 8924–8941 (2015).

56. Pattanayak, V. *et al.* High-throughput profiling of off-target DNA cleavage reveals RNA-programmed Cas9 nuclease specificity. *Nature Biotechnology* **31**, 839–843 (2013).

57. Hsu, P. D. *et al.* DNA targeting specificity of RNA-guided Cas9 nucleases. *Nature Biotechnology* **31**, 827–832 (2013).

58. Fu, Y. *et al.* High-frequency off-target mutagenesis induced by CRISPR-Cas nucleases in human cells. *Nature Biotechnology* **31**, 822–826 (2013).

59. Gilbert, L. *et al.* Genome-Scale CRISPR-Mediated Control of Gene Repression and Activation. *Cell* **159**, 647–661 (2014).

60. Strohkendl, I., Saifuddin, F. A., Rybarski, J. R., Finkelstein, I. J. & Russell, R. Kinetic Basis for DNA Target Specificity of CRISPR-Cas12a. *Molecular Cell* **71**, 816–824.e3 (2018).

61. Fu, Y., Sander, J. D., Reyon, D., Cascio, V. M. & Joung, J. K. Improving CRISPR-Cas nuclease specificity using truncated guide RNAs. *Nature Biotechnology* **32**, 279–284 (2014).

62. Tsai, S. Q. *et al.* GUIDE-seq enables genome-wide profiling of off-target cleavage by CRISPR-Cas nucleases. *Nature Biotechnology* **33**, 187–197 (2015).

63. Kocak, D. D. *et al.* Increasing the specificity of CRISPR systems with engineered RNA secondary structures. *Nature Biotechnology* **37**, 657–666 (2019).

64. Slaymaker, I. M. *et al.* Rationally engineered Cas9 nucleases with improved specificity. *Science* **351**, 84–88 (2016).

65. Kleinstiver, B. P. *et al.* High-fidelity CRISPR–Cas9 nucleases with no detectable genome-wide off-target effects. *Nature* **529**, 490–495 (2016).

66. Bisaria, N., Jarmoskaite, I. & Herschlag, D. Lessons from Enzyme Kinetics Reveal Specificity Principles for RNA-Guided Nucleases in RNA Interference and CRISPR-Based Genome Editing. *Cell Systems* **4**, 21–29 (2017).

67. Maurizi, M. R. Proteases and protein degradation in Escherichia coli. *Experientia* **48**, 178–201 (1992).

68. Flynn, J. M. *et al.* Overlapping recognition determinants within the ssrA degradation tag allow modulation of proteolysis. *Proceedings of the National Academy of Sciences* **98**, 10584–10589 (2001).

69. Purcell, O., Grierson, C. S., Bernardo, M. d. & Savery, N. J. Temperature dependence of ssrA-tag mediated protein degradation. *Journal of Biological Engineering* **6**, 10 (2012).

70. Mizusawa, S. & Gottesman, S. Protein degradation in Escherichia coli: the lon gene controls the stability of sulA protein. *Proceedings of the National Academy of Sciences* **80**, 358–362 (1983).

71. Gur, E. & Sauer, R. T. Degrons in protein substrates program the speed and operating efficiency of the AAA+ Lon proteolytic machine. *Proceedings of the National Academy of Sciences* **106**, 18503–18508 (2009).

72. Lu, P., Vogel, C., Wang, R., Yao, X. & Marcotte, E. M. Absolute protein expression profiling estimates the relative contributions of transcriptional and translational regulation. *Nature Biotechnology* **25**, 117–124 (2007).

73. Ishihama, Y. *et al.* Protein abundance profiling of the Escherichia coli cytosol. *BMC Genomics* **9**, 102 (2008).

74. Arike, L. *et al.* Comparison and applications of label-free absolute proteome quantification methods on Escherichia coli. *Journal of Proteomics* **75**, 5437–5448 (2012).

75. Li, G.-W., Burkhardt, D., Gross, C. & Weissman, J. Quantifying Absolute Protein Synthesis Rates Reveals Principles Underlying Allocation of Cellular Resources. *Cell* **157**, 624–635 (2014).

76. Soufi, B., Krug, K., Harst, A. & Macek, B. Characterization of the E. coli proteome and its modifications during growth and ethanol stress. *Frontiers in Microbiology* **6**, 103 (2015).

77. Csibra, E. & Stan, G.-B. Absolute protein quantification using fluorescence measurements with FPCountR. *Nature Communications* **13**, 6600 (2022).

78. Bintu, L. *et al.* Transcriptional regulation by the numbers: applications. *Current Opinion in Genetics & Development* **15**, 125–135 (2005).

79. Jung, C. *et al.* True equilibrium measurement of transcription factor-DNA binding affinities using automated polarization microscopy. *Nature Communications* **9**, 1605 (2018).

80. Wang, Y., Guo, L., Golding, I., Cox, E. C. & Ong, N. Quantitative Transcription Factor Binding Kinetics at the Single-Molecule Level. *Biophysical Journal* **96**, 609–620 (2009).

81. Nelson, H. C. & Sauer, R. T. Lambda repressor mutations that increase the affinity and specificity of operator binding. *Cell* **42**, 549–558 (1985).

82. Burz, D. S., Beckett, D., Benson, N. & Ackers, G. K. Self-Assembly of Bacteriophage .lambda. cI Repressor: Effects of Single-Site Mutations on the Monomer-Dimer Equilibrium. *Biochemistry* **33**, 8399–8405 (1994).

83. Gatti-Lafranconi, P., Dijkman, W. P., Devenish, S. R. & Hollfelder, F. A single mutation in the core domain of the lac repressor reduces leakiness. *Microbial Cell Factories* **12**, 67 (2013).

84. Niederholtmeyer, H. *et al.* Rapid cell-free forward engineering of novel genetic ring oscillators. *eLife* **4**, e09771 (2015).

85. Tomazou, M., Barahona, M., Polizzi, K. M. & Stan, G.-B. Computational Re-design of Synthetic Genetic Oscillators for Independent Amplitude and Frequency Modulation. *Cell Systems* **6**, 508–520.e5 (2018).

86. Henningsen, J. *et al.* Single Cell Characterization of a Synthetic Bacterial Clock with a Hybrid Feedback Loop Containing dCas9-sgRNA. *ACS Synthetic Biology* **9**, 3377–3387 (2020).

87. Kuo, J., Yuan, R., Sánchez, C., Paulsson, J. & Silver, P. A. Toward a translationally independent RNA-based synthetic oscillator using deactivated CRISPR-Cas. *Nucleic Acids Research* **48**, gkaa557– (2020).

88. Zhang, F. *et al.* Independent control of amplitude and period in a synthetic oscillator circuit with modified repressilator. *Communications Biology* **5**, 23 (2022).

89. Elowitz, M. B. & Leibler, S. A synthetic oscillatory network of transcriptional regulators. *Nature* **403**, 335–338 (2000).

90. Potvin-Trottier, L., Lord, N. D., Vinnicombe, G. & Paulsson, J. Synchronous long-term oscillations in a synthetic gene circuit. *Nature* **538**, 514–517 (2016).

91. Riglar, D. T. *et al.* Bacterial variability in the mammalian gut captured by a single-cell synthetic oscillator. *Nature Communications* **10**, 4665 (2019).

92. Hasunuma, K. & Sekiguchi, M. Replication of plasmid pSC101 inEscherichia coli K12: Requirement fordnaA function. *Molecular and General Genetics MGG* **154**, 225–230 (1977).

93. Cabello, F., Timmis, K. & Cohen, S. N. Replication control in a composite plasmid constructed by in vitro linkage of two distinct replicons. *Nature* **259**, 285–290 (1976).

94. Lutz, R. & Bujard, H. Independent and Tight Regulation of Transcriptional Units in Escherichia Coli Via the LacR/O, the TetR/O and AraC/I1-I2 Regulatory Elements. *Nucleic Acids Research* **25**, 1203–1210 (1997).

95. Peterson, J. & Phillips, G. J. New pSC101-derivative cloning vectors with elevated copy numbers. *Plasmid* **59**, 193–201 (2008).

96. Thompson, M. G. *et al.* Isolation and characterization of novel mutations in the pSC101 origin that increase copy number. *Scientific Reports* **8**, 1590 (2018).

97. Shao, B. *et al.* Single-cell measurement of plasmid copy number and promoter activity. *Nature Communications* **12**, 1475 (2021).

98. Meyer, A. J., Segall-Shapiro, T. H., Glassey, E., Zhang, J. & Voigt, C. A. Escherichia coli "Marionette" strains with 12 highly optimized small-molecule sensors. *Nature Chemical Biology* **15**, 196–204 (2019).

99. Beerli, R. R., Segal, D. J., Dreier, B. & Barbas, C. F. Toward controlling gene expression at will: Specific regulation of the erbB-2/HER-2 promoter by using polydactyl zinc finger proteins constructed from modular building blocks. *Proceedings of the National Academy of Sciences* **95**, 14628–14633 (1998).

100. Haynes, K. A. & Silver, P. A. Synthetic Reversal of Epigenetic Silencing*. *Journal of Biological Chemistry* **286**, 27176–27182 (2011).

101. Zinselmeier, M. H. *et al.* Optimized dCas9 Programmable Transcription Activators for Plants. *bioRxiv* 2022.06.10.495638 (2022).

102. Cameron, D. E. & Collins, J. J. Tunable protein degradation in bacteria. *Nature Biotechnology* **32**, 1276–1281 (2014).

103. Wang, Y. M. *et al.* Single-molecule studies of repressor–DNA interactions show long-range interactions. *Proceedings of the National Academy of Sciences* **102**, 9796–9801 (2005).

104. Qi, L. *et al.* Repurposing CRISPR as an RNA-Guided Platform for Sequence-Specific Control of Gene Expression. *Cell* **152**, 1173–1183 (2013).

105. Nielsen, A. A. & Voigt, C. A. Multi-input CRISPR/Cas genetic circuits that interface host regulatory networks. *Molecular Systems Biology* **10**, 763 (2014).

106. Anderson, D. A. & Voigt, C. A. Competitive dCas9 binding as a mechanism for transcriptional control. *Molecular Systems Biology* **17**, e10512 (2021).

107. Jones, D. L. *et al.* Kinetics of dCas9 target search in Escherichia coli. *Science* **357**, 1420–1424 (2017).

108. Zhang, S. & Voigt, C. A. Engineered dCas9 with reduced toxicity in bacteria: implications for genetic circuit design. *Nucleic Acids Research* **46**, gky884– (2018).

109. Stricker, J. *et al.* A fast, robust and tunable synthetic gene oscillator. *Nature* **456**, 516–519 (2008).

110. Scott, M., Gunderson, C. W., Mateescu, E. M., Zhang, Z. & Hwa, T. Interdependence of Cell Growth and Gene Expression: Origins and Consequences. *Science* **330**, 1099–1102 (2010).

111. Ceroni, F. *et al.* Burden-driven feedback control of gene expression. *Nature Methods* **15**, 387–393 (2018).

112. Qian, Y., Huang, H.-H., Jiménez, J. I. & Vecchio, D. D. Resource Competition Shapes the Response of Genetic Circuits. *ACS Synthetic Biology* **6**, 1263–1272 (2017).

113. Kaplan, S., Bren, A., Dekel, E. & Alon, U. The incoherent feed-forward loop can generate non-monotonic input functions for genes. *Molecular Systems Biology* **4**, 203–203 (2008).

114. Barajas, C., Huang, H.-H., Gibson, J., Sandoval, L. & Vecchio, D. D. Feedforward growth rate control mitigates gene activation burden. *Nature Communications* **13**, 7054 (2022).

115. Higham., D. J. An algorithmic introduction to numerical simulation of stochastic differential equations. *SIAM Review* **43**, 525–546 (2001).

116. Gallant, J., Margason, G. & Finch, B. On the turnover of ppGpp in Escherichia coli. *The Journal of biological chemistry* **247**, 6055–8 (1972).

117. Friesen, J. D., Fiil, N. P. & Meyenburg, K. v. Synthesis and turnover of basal level guanosine tetraphosphate in Escherichia coli. *The Journal of biological chemistry* **250**, 304–9 (1975).

118. Fiil, N. P., Willumsen, B. M., Friesen, J. D. & Meyenburg, K. v. Interaction of alleles of therelA, relC andspoT genes inEscherichia coli: Analysis of the interconversion of GTP, ppGpp and pppGpp. *Molecular and General Genetics MGG* **150**, 87–101 (1977).

119. Moon, T. S., Lou, C., Tamsir, A., Stanton, B. C. & Voigt, C. A. Genetic programs constructed from layered logic gates in single cells. *Nature* **491**, 249–253 (2012).

120. Zaslaver, A. *et al.* Invariant Distribution of Promoter Activities in Escherichia coli. *PLoS Computational Biology* **5**, e1000545 (2009).

121. Scott, M., Klumpp, S., Mateescu, E. M. & Hwa, T. Emergence of robust growth laws from optimal regulation of ribosome synthesis. *Molecular Systems Biology* **10**, 747 (2014).

122. Nielsen, A. A. K. *et al.* Genetic circuit design automation. *Science* **352**, aac7341–aac7341 (2016).
