# Supplementary material for: A blueprint for a synthetic genetic feedback optimizer
Source: Nat Commun. 2023 May 3;14:2554. doi: 10.1038/s41467-023-37903-0 (PMC10156725; doi:10.1038/s41467-023-37903-0)
Supplement: Supplementary file 3 — Reporting Summary [file 41467_2023_37903_MOESM3_ESM.pdf]

nature portfolio

Corresponding author(s): Andras Gyorgy

Last updated by author(s): Mar 27, 2023

Reporting Summary

Nature Portfolio wishes to improve the reproducibility of the work that we publish. This form provides structure for consistency and transparency in reporting. For further information on Nature Portfolio policies, see our Editorial Policies and the Editorial Policy Checklist.

Statistics

For all statistical analyses, confirm that the following items are present in the figure legend, table legend, main text, or Methods section.

n/a	Confirmed	
☐	☒	The exact sample size (n) for each experimental group/condition, given as a discrete number and unit of measurement
☒	☐	A statement on whether measurements were taken from distinct samples or whether the same sample was measured repeatedly
☒	☐	The statistical test(s) used AND whether they are one- or two-sided<br>*Only common tests should be described solely by name; describe more complex techniques in the Methods section.*
☒	☐	A description of all covariates tested
☒	☐	A description of any assumptions or corrections, such as tests of normality and adjustment for multiple comparisons
☐	☒	A full description of the statistical parameters including central tendency (e.g. means) or other basic estimates (e.g. regression coefficient) AND variation (e.g. standard deviation) or associated estimates of uncertainty (e.g. confidence intervals)
☒	☐	For null hypothesis testing, the test statistic (e.g. F, t, r) with confidence intervals, effect sizes, degrees of freedom and P value noted<br>*Give P values as exact values whenever suitable.*
☒	☐	For Bayesian analysis, information on the choice of priors and Markov chain Monte Carlo settings
☒	☐	For hierarchical and complex designs, identification of the appropriate level for tests and full reporting of outcomes
☒	☐	Estimates of effect sizes (e.g. Cohen's d, Pearson's r), indicating how they were calculated

Our web collection on statistics for biologists contains articles on many of the points above.

Software and code

Policy information about availability of computer code

Data collection	The manuscript does not rely on custom mathematical algorithms or software. Simulation data were generated as described in the Methods by considering the models detailed in the Supplementary Information using built-in MATLAB (version R2022b) functions. The MATLAB code for implementing and deploying the optimizer for the examples featured in the manuscript, along with instructions on how to modify it for other application examples, are publicly available at https://github.com/qbionet/genetic-optimizer. Additional information is available from the corresponding author upon request.
Data analysis	Simulation data were analyzed and plotted using MATLAB (version R2022b). Figures were created using Adobe Illustrator (version 26.5). Overleaf (version v2) online LaTeX editor was used to prepare and compile the manuscript.

For manuscripts utilizing custom algorithms or software that are central to the research but not yet described in published literature, software must be made available to editors and reviewers. We strongly encourage code deposition in a community repository (e.g. GitHub). See the Nature Portfolio guidelines for submitting code & software for further information.

Data

Policy information about availability of data

All manuscripts must include a data availability statement. This statement should provide the following information, where applicable:

- Accession codes, unique identifiers, or web links for publicly available datasets
- A description of any restrictions on data availability
- For clinical datasets or third party data, please ensure that the statement adheres to our policy

Source data are provided with this paper.

Human research participants

Policy information about studies involving human research participants and Sex and Gender in Research.

Reporting on sex and gender	N/A
Population characteristics	N/A
Recruitment	N/A
Ethics oversight	N/A

Note that full information on the approval of the study protocol must also be provided in the manuscript.

Field-specific reporting

Please select the one below that is the best fit for your research. If you are not sure, read the appropriate sections before making your selection.

☒ Life sciences ☐ Behavioural & social sciences ☐ Ecological, evolutionary & environmental sciences

For a reference copy of the document with all sections, see nature.com/documents/nr-reporting-summary-flat.pdf

Life sciences study design

All studies must disclose on these points even when the disclosure is negative.

Sample size	Statistical methods were not used to predetermine sample sizes. Instead, sample sizes were selected to ensure that closed loop performance is evaluated when considering a diverse array of initial conditions and stochastic noise profiles.
Data exclusions	No data were excluded from the analysis.
Replication	Numerical simulations were performed repeatedly to ensure that results are consistent across trials considering randomly selected initial conditions and stochastic noise profiles. All attempts at replication were successful.
Randomization	Initial conditions and stochastic noise profiles were generated randomly to ensure that closed loop performance is evaluated when considering a diverse array of numerical simulations.
Blinding	Blinding was not not applicable: success metric for our controller design was pre-determined (closed loop performance relative to the optimum).

Reporting for specific materials, systems and methods

We require information from authors about some types of materials, experimental systems and methods used in many studies. Here, indicate whether each material, system or method listed is relevant to your study. If you are not sure if a list item applies to your research, read the appropriate section before selecting a response.

Materials & experimental systems

n/a	Involved in the study
☒	☐ Antibodies
☒	☐ Eukaryotic cell lines
☒	☐ Palaeontology and archaeology
☒	☐ Animals and other organisms
☒	☐ Clinical data
☒	☐ Dual use research of concern

Methods

n/a	Involved in the study
☒	☐ ChIP-seq
☒	☐ Flow cytometry
☒	☐ MRI-based neuroimaging
